# Practical Real-Time Quaking-Induced Conversion for Detecting Classical Bovine Spongiform Encephalopathy and Classical and Atypical Scrapie Prions

**DOI:** 10.3390/pathogens15030333

**Published:** 2026-03-20

**Authors:** Akio Suzuki, Kazuhei Sawada, Taku Nakashima, Toyotaka Sato, Kohtaro Miyazawa, Yuichi Matsuura, Keigo Ikeda, Yoshifumi Iwamaru, Motohiro Horiuchi

**Affiliations:** 1 Laboratory of Veterinary Hygiene, Faculty of Veterinary Medicine, Graduate School of Infectious Diseases, Hokkaido University, Kita 18, Nishi 9, Kita-ku, Sapporo 060-0818, Hokkaido, Japan; susan@vetmed.hokudai.ac.jp (A.S.); sato.t@vetmed.hokudai.ac.jp (T.S.); 2 One Health Research Center, Hokkaido University, Kita 18, Nishi 9, Kita-ku, Sapporo 060-0818, Hokkaido, Japan; 3 Division of Infectious Animal Diseases, National Institute of Animal Health, Kannondai 3-1-5, Tsukuba 305-0856, Ibaraki, Japan; miyazawa.kohtaroh090@naro.go.jp (K.M.); matsuura.yuichi852@naro.go.jp (Y.M.); ikeda.keigo814@naro.go.jp (K.I.); iwamaru.yoshifumi549@naro.go.jp (Y.I.); 4 Veterinary Research Unit, International Institute for Zoonosis Control, Hokkaido University, Kita 20, Nishi 10, Kita-ku, Sapporo 060-0818, Hokkaido, Japan

**Keywords:** prion, transmissible spongiform encephalopathy, C-BSE, scrapie, RT-QuIC, lipid extraction, 2-butanol/methanol

## Abstract

Real-time quaking-induced conversion (RT-QuIC) is highly sensitive for prion detection; however, inhibitory factors present in tissue homogenates readily interfere with the assay. We previously reported that recombinant cervid prion protein (rCerPrP) enabled the establishment of practical RT-QuIC for detecting chronic wasting disease and atypical bovine spongiform encephalopathy (BSE) prions, i.e., detecting low levels of prions in high concentration of brain tissue homogenates. Accordingly, the present study aimed to establish RT-QuIC for detecting classical BSE (C-BSE) and classical and atypical scrapie (C- and A-scrapie, respectively). A single-step lipid extraction using a 3:1 mixture of 2-butanol and methanol was effective as a pretreatment to remove inhibitors from brain homogenates. Among three rPrPs extensively evaluated, recombinant sheep PrP (rShPrP) was the most suitable substrate for practical detection of C-BSE prions. rCerPrP-173S/177N and rCerPrP-98S/173S/177N, which carry sheep-type amino acid substations at codons 173 and 177 and at codons 98, 173, and 177, showed excellent performance for detecting C-scrapie prions. Moreover, rCerPrP-98S/173S/177N, but not rCerPrP-173S/177N, was identified as an optimal substrate for detecting A-scrapie prions. These results suggested that combining inhibitor-removal pretreatment with the optimization of rPrP substrate for each animal prions further enhanced of RT-QuIC performance.

## 1. Introduction

Prion diseases are fatal neurodegenerative disorders that include Creutzfeldt–Jakob disease (CJD), Gerstmann–Sträussler–Scheinker syndrome, and fatal insomnia in humans, as well as scrapie in sheep and goats, chronic wasting disease (CWD) in cervids, and bovine spongiform encephalopathy (BSE) [1]. The causative agents, prions, consist primarily of an abnormal isoform of a prion protein (PrP^Sc^). PrP^Sc^ is generated from the host-encoded cellular isoform of PrP (PrP^C^) through interaction with pre-existing PrP^Sc^ and subsequent conformational conversion from α-helix-rich PrP^C^ to β-structure-rich PrP^Sc^. Following prion infection, PrP^Sc^ is generated and accumulates in the central nervous system (CNS) over a prolonged incubation period. The accumulation of PrP^Sc^ in the CNS represents a defining pathological feature of prion diseases.

Animal prions spread horizontally via prion-contaminated tissues and environment. Additionally, prions may emerge spontaneously in aged animals and cause sporadic diseases [2]. When prions are shed into the environment via carcasses, body fluids, and excreta from prion-infected animals, they can persist for long periods in soil pastures, enabling sustained horizontal transmission [3,4,5,6,7]. The global incidence of classical BSE (C-BSE), which infects humans and causes variant CJD [8], is now largely controlled. However, prion properties can change during interspecies transmission [9,10,11,12]; therefore, the emergence of novel animal prions with zoonotic potential cannot be excluded. Furthermore, L-type atypical BSE (L-BSE) prions are considered to have zoonotic potential because they infect primates and gene-engineered mice expressing human *Prnp*, and low levels of prions have been detected in the edible tissues of L-BSE-infected cattle [13,14,15,16]. Consequently, simple yet reliable methods for continuous monitoring of animal prion diseases are required to enable early responses to potentially emerging and re-emerging animal prion diseases and ensure the safety of animal-derived products.

Real-time quaking-induced conversion (RT-QuIC) [17] was developed for prion detection and has been applied for detecting low levels of prions in tissue homogenates and body fluids. RT-QuIC measures the amyloid seeding activity of PrP^Sc^ aggregates, and amyloid fibrils generated during the reaction are monitored using the amyloid-binding dye thioflavin T (ThT). RT-QuIC offers substantially higher detection sensitivity relative to immunobiochemical and immunohistochemical methods [13,18,19], which have served as the gold standard for PrP^Sc^ detection. However, this method is readily interfered with inhibitory factors in tissue homogenates and body fluids [20,21,22], limiting the detection of low levels of prions in high concentrations of tissue homogenates. Although the removal of inhibitory factors through PrP^Sc^ enrichment using immunoprecipitation, iron oxide beads, and phosphotungstic acid, as well as lipid removal with alcohol extraction, can partially reduce the interference of RT-QuIC assay [20,21,23,24], further optimization remains necessary for the practical detection of animal prions.

We previously demonstrated that the detection of CWD and atypical BSE prions by RT-QuIC using recombinant cervid PrP (rCerPrP) was minimally affected by high concentrations of normal brain homogenate (NBH) [25]. These findings suggest that, in addition to the removal of inhibitory factors, selecting an appropriate rPrP substrate is critical for improving RT-QuIC performance. Nevertheless, using RT-QuIC for the detection of C-BSE and classical and atypical scrapie (C- and A-scrapie, respectively) prions still requires improvement to achieve practical utility ([25]; our preliminary experiment), namely the reliable detection of low levels of prions in high concentrations of tissue homogenates and/or body fluids. Thus, this study aims to establish an RT-QuIC suitable for the practical detection of C-BSE and C- and A-scrapie prions by integrating a single-step lipid removal method with rPrP substrate optimization.

## 2. Materials and Methods

### 2.1. Reagents and Chemicals

The solvent used for lipid extraction included 2-butanol (BuOH; Fujifilm Wako Pure Chemical Corporation, Osaka, Japan), ethanol (EtOH; Fujifilm Wako Pure Chemical Corporation), and methanol (MeOH; Kanto Chemical Co., Inc., Tokyo, Japan). Phosphatidylcholine (PC), phosphatidylethanolamine (PE), and phosphatidylserine (PS) were purchased from Funakoshi, Japan, and sphingomyelins (SM; Sigma-Aldrich, St. Louis, MO, USA) were used as phospholipids in the brain.

### 2.2. Brain Materials

Brain tissues from 22L, Chandler, and Obihiro strain-infected Jcl:ICR mice at the terminal stage of the disease were used as sources of mouse-adapted scrapie prions, whereas those from age-matched, mock-infected Jcl:ICR mice were used as sources of negative controls and seed diluents for RT-QuIC. All mice were housed at the Faculty of Veterinary Medicine, Hokkaido University, in an AAALAC International-accredited facility, following protocols approved by the Institutional Animal Care and Use Committee, Hokkaido University (approval No. 23-0059). Ten percent brain homogenates of three C-BSE-affected cattle [26] at 67–83 months old, which were disclosed through BSE screening in Japan, and six C-scrapie-affected sheep [27] at 16–48 months old—four were naturally occurred cases in Japan, while the remaining two were experimental infections—were used as seeds, and the corresponding species of prion-negative brain homogenates were used as negative controls. The brain homogenates of A-scrapie-affected sheep, experimentally inoculated with the brain homogenates of A-scrapie-affected sheep from the UK, were prepared at the National Institute of Animal Health, Japan. Information on the animals used in this study is summarized in Table S1. The brains were homogenized with phosphate-buffered saline (PBS) at 10% or 20% and stored at −80 °C until use. Immediately before use, 2% of brain homogenates were freshly prepared in PBS. The levels of proteinase K-resistant PrP (PrP-res) in each brain homogenate are shown in Table S1.

### 2.3. Expression and Purification of rPrPs

Expression plasmids encoding full-length mouse and hamster rPrPs (rMoPrP and rHaPrP, respectively; amino acid [aa] 23–231) were kindly provided by Dr. Atarashi R., Miyazaki University, Japan. A plasmid encoding full-length bank vole rPrP (rBvPrP: aa 23–230) was kindly provided by Dr. Caughey B., National Institute of Health, USA. Constructions of expression plasmids for sheep rPrP (rShPrP) carrying alleles encoding A, R, and Q at codons 136, 154, and 171 (genotype; A_136_R_154_Q_171_), respectively, and rCerPrP-173S/177N, which carries two aa substitutions at codons 173 (Asn to Ser) and 177 (Thr to Asn) in wild-type rCerPrP (genotype: G_96_M_132_S_225_Q_226_), has been described previously [25]. The gene coding rCerPrP-98S/173S/177N, containing three aa substitutions at codons 98, 173, and 177 in wild-type rCerPrP, was generated by replacing the Thr98 codon of CerPrP (ACC) with the Ser98 codon from ShPrP (TCC) using assembly PCR from the plasmid containing rCerPrP-173S/177N gene as well as the primers CerT98S-F (5′-CAAGGTGGTTCCCACAGTGGAA-3′) and CerT98S-R (5′-CTGACTGTGGGAACCACCTTGACC-3′), as reported previously [25]. The expression of rPrPs was performed as described previously [25]. Only four aa differences existed between ShPrP and CerPrP at codons 98 (Ser/Thr), 173 (Ser/Asn), 177 (Asn/Thr), and 208 (Ile/Met); therefore, three rPrPs shared high primary sequence similarity.

### 2.4. Purification of rPrPs

The purification of rPrPs was performed as described previously [25]. The rPrP was refolded using a linear gradient of 6 to 0 M guanidine hydrochloride (GdnHCl) in a refolding buffer (100 mM sodium phosphate [pH 8.0], 10 mM tris) over 22 column volume (CV) at a 0.55 mL/min flow rate using ÄKTAexplore 10S system (GE Healthcare), and then eluted by a linear gradient of 0 to 500 mM imidazole in 10 mM tris (pH 5.8) over 3 CV at a 1.0 mg/min flow rate. The fractions in the main peak were pooled and dialyzed against ultra-pure water. After the filtration and determination of rPrP concentration, 1 mL aliquots of the rPrPs were stored at −30 °C until use, and the thawed rPrPs were used up within 2–3 freeze–thaw cycles.

### 2.5. RT-QuIC Assays

RT-QuIC assays were carried out using a 100 μL final reaction mixture per well that included 95 μL of reaction mixture and 5 μL of seed solution. A reaction mixture (95 μL) containing 10 mM phosphate buffer (pH 7.4), 500 mM sodium chloride, 10 μM ThT, 1 mM ethylendiaminetetraacetic acid, 0.001% sodium dodecyl sulfate, and 100 μg/mL rPrP as a final concentration was added to the wells of a 96-well optical bottom plate (Nunc, Roslilde, Denmark). Brain homogenates (2%) from prion-affected animals (Figure S2) were serially diluted from 0.2% to 0.000002% (corresponding to final dilution of 10^−4^ to 10^−9^ dilutions, respectively) in PBS or 2% NBH from the corresponding species, and 5 μL of each dilution was used as a seed. Indeed, 10^−4^ meant that the concentration of brain homogenates used as seeds in the final reaction mixture was 0.01%. RT-QuIC assays with rBvPrP, rHaPrP, and rMoPrP were performed under the reaction conditions using an Infinite F200 microplate reader (TECAN, Männedorf, Switzerland) at 37 °C for 360 cycles as the RT-QuIC assays using these substrates have been established as previously described [25]. Each cycle comprised 30 s of orbital shaking at 432 rpm and 30 s of settling, and then a measurement of fluorescence with excitation at 430 nm and emission at 485 nm with a manually selected gain of 55 was obtained. RT-QuIC assays using rShPrP, rCerPrP-173S/177N, and rCerPrP-98S/173S/177N were newly established using a BMG FLUOStar Omega microplate reader (Reader Control v5.70) (BMG Labtech, Berlin, Germany) conducted at 37 °C for 360 cycles—this provided a superior signal-to-noise ratio and, as a consequence, the plate reader could reduce the appearance of atypical waves observed previous studies [28]. Each cycle comprised 30 s of orbital shaking at 800 rpm or double orbital shaking at 700 rpm for the rShPrP and rCerPrP mutants, respectively, followed by 30 s of settling and fluorescence measurement every 600 s with an adjusted gain of 1900–1920. For RT-QuIC assays with commercially available lipids, PC, PE, PS, and SM were dissolved in a mixture of BuOH and MeOH (BuMe; 5:1) at 10–25 mg/mL and stored at −30 °C. Lipid solutions were diluted in PBS to concentrations equivalent to 2% NBH based on the lipid composition of the human brain [29], 0.002% for PC, 0.003% for PE, 0.0003% for PS, and 0.0013% for SM, or to total phospholipids concentration of 0.007%, and used as seed diluents.

### 2.6. Single-Step Lipid Extraction

The total lipids were extracted from seeds diluted in 2% NBH from mouse, bovine, or sheep using an alcohol-based protein precipitation method [30,31,32] with EtOH or a 3:1 mixture of BuOH and MeOH (BuMe [3:1]) [33] with subtle modifications. Briefly, prion-infected 2% brain homogenates diluted to 10^−4^–10^−9^ in the corresponding 2% NBH were mixed with 40 volumes of EtOH or BuMe (3:1) and incubated for 10 min at room temperature. Samples were then centrifuged at 20,000× *g* at 20 °C for 10 min. After discarding the supernatants, pellets were resuspended in the original volumes of PBS by sonication at 35% amplitude using a Digital Sonifier Model 450 with a cup-horn (Branson, CT, USA) for 2 min.

### 2.7. Determination of Positive Reaction

The positive wells were determined as described in the previous study [25]. The endpoint was determined as the highest seed dilution that gave a positive reaction in three independent experiments or one experiment with triplicate wells. If a low dilution turned out to be negative and a positive well subsequently appeared in a higher dilution, the negative result at the lower dilution was evaluated as the endpoint. In addition to these determination criteria, we set an assay endpoint for the lag phase. Spontaneous reactions observed after 50 h in 20 wells out of 747 wells were used as the negative control for detection using rShPrP and mutants of rCerPrP, and the mean time at which spontaneous reactions were detected was calculated as 55.8 h; therefore, we set an assay endpoint at 55 h. On the other hand, spontaneous reactions rarely occurred in the cases of RT-QuIC using rMoPrP, rBvPrP, and rHaPrP, so the assay endpoint was set at 60 h. Mean and standard deviation (SD) were calculated using wells that were determined to be positive out of the total numbers of wells.

### 2.8. Statistical Analyses

The statistical analyses of the lag phase were carried out as follows. The normality of data distribution and homogeneity of variances were assessed using the Shapiro–Wilk test and *F*-test, respectively. When the Shapiro–Wilk test indicated a non-normal distribution (*p* < 0.05) for either PBS-diluted or BuMe-extracted samples, Mann-Whiteney’s *U*-test was applied; otherwise, Welch’s *t*-test was used.

## 3. Results

### 3.1. Establishment of a Model RT-QuIC System Using Mouse-Adapted Prion Strains

To establish a model RT-QuIC system for evaluating inhibitory factors, we examined the reactivity of full-length rodent rPrPs to 22L, Chandler, and Obihiro mouse-adapted prion strains in the absence and presence of NBH (Table S2). Without NBH, rMoPrP and rBvPrP detected all three strains in more than 10^−8^ dilution (10^−8^ or <10^−9^, diluent: PBS), whereas rHaPrP exhibited lower reactivity, with the lag phases prolonged by 5.7–22.5 h compared with rMoPrP and rBvPrP at 10^−4^ and 10^−5^ dilutions for Chandler and Obihiro strains and with endpoints reduced by 1–2 logs for all strains. In contrast, in the presence of 0.1% NBH, all RT-QuIC reactions were completely inhibited, except for the rMoPrP–22L combination, which retained a detection endpoint of 10^−5^ with a significantly prolonged lag phase (Table S2). Because the rMoPrP–22L combination produced the highest detection endpoint under both conditions, this combination was selected for use in subsequent experiments.

### 3.2. Application of the Single-Step Lipid Extraction with Butanol/Methanol to RT-QuIC

Given that lipids interfere with RT-QuIC reactions [20], lipid removal is required for practical RT-QuIC detection of animal prions, particularly for low levels of prions in high concentrations of CNS homogenates. First, we assessed the inhibitory effect of lipid and protein fractions. Lipids were extracted from 10% NBH by one, three, and six repeated EtOH extractions, as described in the Supplementary Information [20]. Lipid-containing supernatants were evaporated and reconstituted in EtOH, whereas protein-containing precipitates were reconstituted in PBS. After six repeated extractions, the two fractions were mixed to reconstitute 2% NBH; proteins, including PrP^C^, were then recovered in precipitates but were undetectable in supernatants following repeated EtOH extraction (Figure S3A,B). After six consecutive EtOH extractions, the reconstituted supernatant fraction and reconstituted NBH completely inhibited RT-QuIC reactions, whereas the precipitate fraction did not, indicating that EtOH-extracted lipids, rather than proteins, inhibited the reaction (Figure S2C). One to three EtOH extraction cycles were insufficient to restore RT-QuIC to the levels observed in PBS-diluted samples (Figure S3D).

Alcohol-based protein precipitation has been used for lipid extraction in liquid chromatography-mass spectrometry [31,32], and a 3:1 BuMe mixture was found to be highly efficient [33]; therefore, we applied this method for single-step lipid extraction in CNS sample preparation (Table 1). The single-step BuMe (3:1) extraction restored the detection endpoint to 10^−9^ (Table 1, NBH/BuMe [3:1]). The endpoint was improved to almost the same level as that observed without NBH, although the lag phases at 10^−4^–10^−6^ dilutions were prolonged by 7.4–12.8 h compared with those of PBS-diluted samples (PBS; Table 1). EtOH extraction also improved RT-QuIC assays, but it was less effective than BuMe (3:1) extraction; that is, single and double EtOH extractions improved the detection endpoints to 10^−6^ and 10^−7^, respectively (Table 1), whereas additional extraction cycles would be required to match a single BuMe (3:1) extraction. These results indicated that single-step BuMe (3:1) extraction could be applicable to pretreatment of brain tissue homogenates for RT-QuIC. We confirmed that a single-step BuMe (3:1) extraction retained proteins, including PrP^C^, in the precipitate fraction (Figure S4).

### 3.3. Practical RT-QuIC Method for Detecting C-BSE and Scrapie Prions

RT-QuIC for detecting C-BSE and C- and A-scrapie prions requires improvement for practical application ([25]; our preliminary experiment). Among 15 rPrPs previously tested [25], rCerPrP-173S/177N was identified as a candidate substrate for RT-QuIC detecting C-BSE and scrapie prions. Additionally, rCerPrP-98S/173S/177N and rShPrP were included because substitutions of CerPrP at codons Thr98 and Met208 to Ser and Ile, the corresponding aa residues of rShPrP, respectively, enhanced the cross-species protein misfolding cyclic amplification (PMCA) reactions between cervid and sheep prions [34].

Naturally occurring scrapie and BSE cases differed in PrP^Sc^ levels and amyloid seeding activity owing to the variation in disease stages. Additionally, limited case numbers in Japan have resulted in variability in the sampled brain regions and storage conditions, potentially influencing RT-QuIC outcomes. Therefore, RT-QuIC performance was not evaluated solely via endpoints in PBS-diluted samples. Instead, three criteria were applied: (i) a detection endpoint after BuMe extraction as an indicator of practical analytical sensitivity; (ii) endpoint recovery defining the ratio of BuMe-extracted to PBS-diluted endpoints; and (iii) lag-phase prolongation after BuMe extraction at low dilutions (e.g., 10^−4^ and 10^−5^), reflecting RT-QuIC assay efficiency [15].

Table 2 shows the results of RT-QuIC for C-BSE detection. Three Japanese C-BSE cases (WA, KUS, and TE) were diluted in PBS or NBH, and NBH-diluted samples were subjected to BuMe (3:1) extraction. The mean detection endpoints after BuMe extraction of WA, KUS, and TE using rCerPrP-173S/177N, rCerPrP-98S/173S/177N, and rShPrP were 10^−6.5^, 10^−6.0^, and 10^−6.3^, respectively b. With rCerPrP-173S/177N, post-extraction endpoints for WA, KUS, and TE decreased by 10^2^, 10^1^, and 10^1^, respectively. Those with rCerPrP-98S/173S/177N also decreased by 10^2^, 10^1^, and 10^1^. In contrast, an endpoint reduction was not observed with rShPrP except for the endpoint for WA (Table 2). The mean endpoint recoveries after BuMe extraction were 10^−1.3^, 10^−1.0^, and 10^0.0^ for rCerPrP-173S/177N, rCerPrP-98S/173S/177N, and rShPrP, respectively. Significant lag-phase prolongation at 10^−4^ or 10^−5^ dilutions was observed for rCerPrP-173S/177N and rCerPrP-98S/173S/177N after BuMe extraction, but not for rShPrP. These results indicated that rShPrP was the most suitable substrate among those tested for practical C-BSE detection. Although detection endpoints after BuMe extraction using rCerPrP-173S/177N and rCerPrP-98S/173S/177N were comparable to those obtained with rShPrP, the endpoint recovery after BuMe extraction was slightly reduced and the lag phases were prolonged.

Table 3 shows the results of RT-QuIC for C-scrapie detection. Six C-scrapie cases, including two experimental infections (B3 and G1) and four naturally occurring Japanese cases (Y5, KH2, S2 and S3), were used. The mean detection endpoints after BuMe extraction using rCerPrP-173S/177N, rCerPrP-98S/173S/177N, and rShPrP were 10^−6.5^, 10^−6.8^, and 10^−5.7^, respectively, and the corresponding mean endpoint recoveries were 10^−0.8^, 10^−0.7^, and 10^−0.8^, respectively. Although lag-phase prolongation occurred for all three substrates in several reactions, rCerPrP-173S/177N and rCerPrP-98S/173S/177N outperformed rShPrP based on the detection endpoints and endpoint recovery following BuMe extraction. Table 4 shows the result of RT-QuIC for A-scrapie detection. Despite the availability of only one A-scrapie sample, rCerPrP-173S/177N was clearly unsuitable as a substrate for detecting A-scrapie. Both rCerPrP-98S/173S/177N and rShPrP detected PBS-diluted A-scrapie with high sensitivity; however, rCerPrP-98S/173S/177N showed superior performance based on the detection endpoints and recovery.

**Figure 1:**
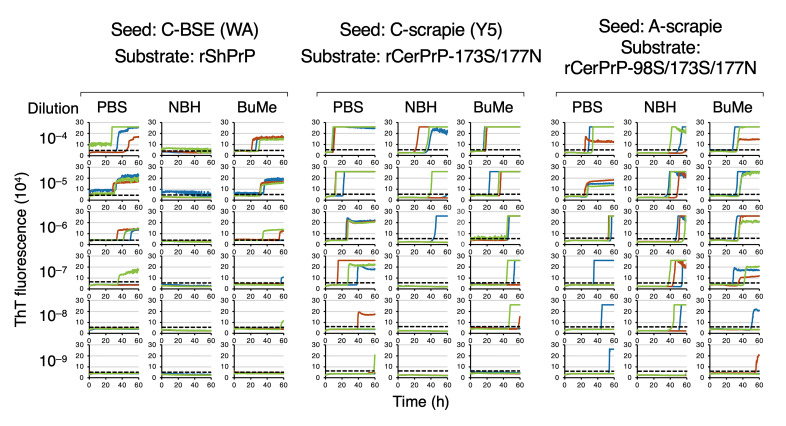
RT-QuIC detection of C-BSE, C-scrapie, and A-scrapie. Representative RT-QuIC amplification curves for detection of each prion type using selected rPrP substrates. Seeds were serially diluted (10^−4^–10^−9^) with PBS or 2% corresponding normal brain homogenate (NBH; cattle brain for C-BSE and sheep brain for scrapie). NBH-diluted seeds were subjected to lipid extraction using BuMe (3:1). Red, blue, and green colors indicate an individual well triplicated in one experiment. Dotted lines indicate reaction thresholds calculated as the mean ThT fluorescence intensity plus 5 × SD of negative control wells.

**Figure 2:**
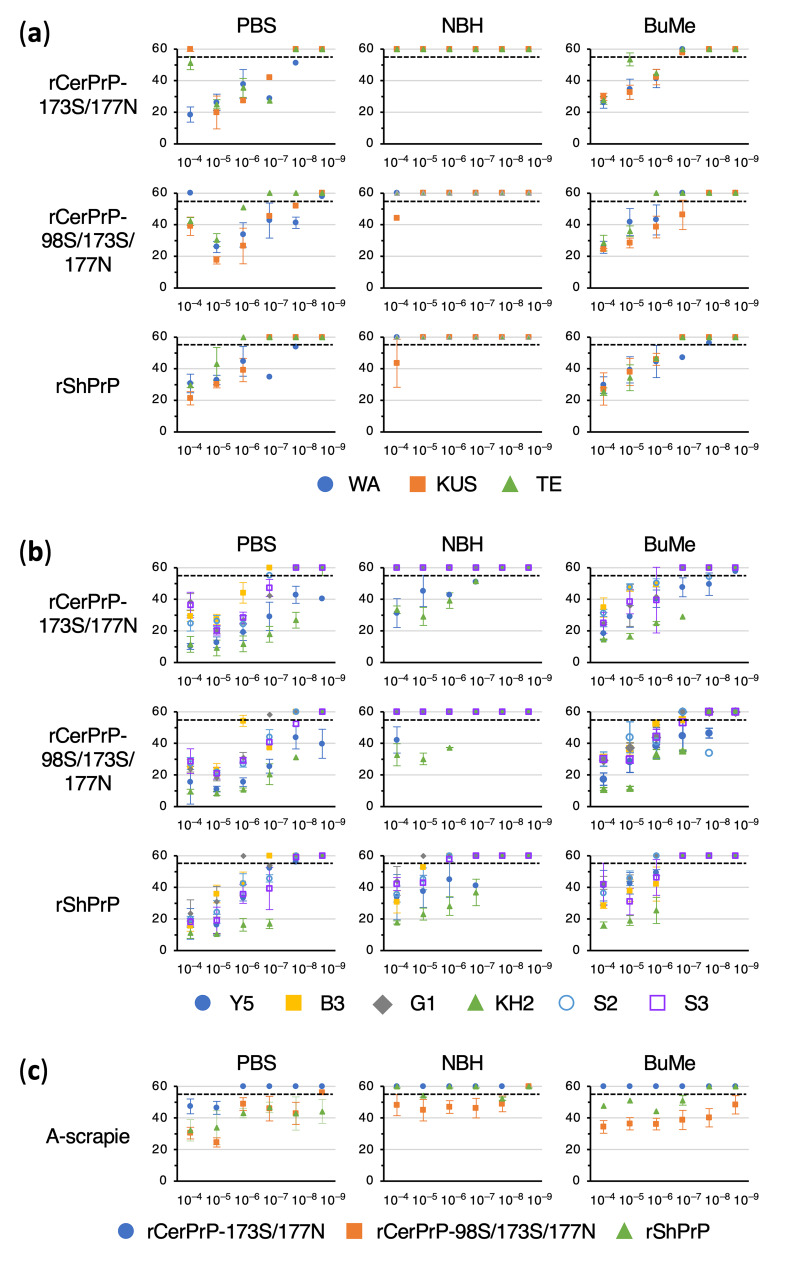
Summary of the lag phases for the detection of C-BSE, C-scrapie, and A-scrapie. Lag phases for the detection of C-BSE (**a**), C-scrapie (**b**), and (**c**) A-scrapie. All lag phases determined with rCerPrP-1773S/177N, rCerPrP-98S/173S/177N, and rShPrP were plotted against seed dilutions with PBS and NBH, as well as seeds after lipid extraction using BuMe (3:1) (PBS, NBH, and BuMe). Dashed lines indicate an assay endpoint set at 55 h. All reactions were measured up to 60 h and lag phases were plotted up to 60 h. The first point that exceeds the assay endpoint indicates the detection endpoint.

Figure 1 shows the representative amplification curves for C-BSE (WA), C-scrapie (Y5), and A-scrapie using the optimized rPrP substrates. The detection of WA and Y5 was markedly inhibited by NBH, whereas detection endpoints were restored after BuMe extraction. For A-scrapie, NBH had a limited impact on the endpoints but caused lag-phase prolongation, which was reduced by BuMe extraction. To visualize the prolongation of the lag phases and the recoveries after BuMe extraction, lag phases are plotted on graphs in Figure 2.

**Figure 3:**
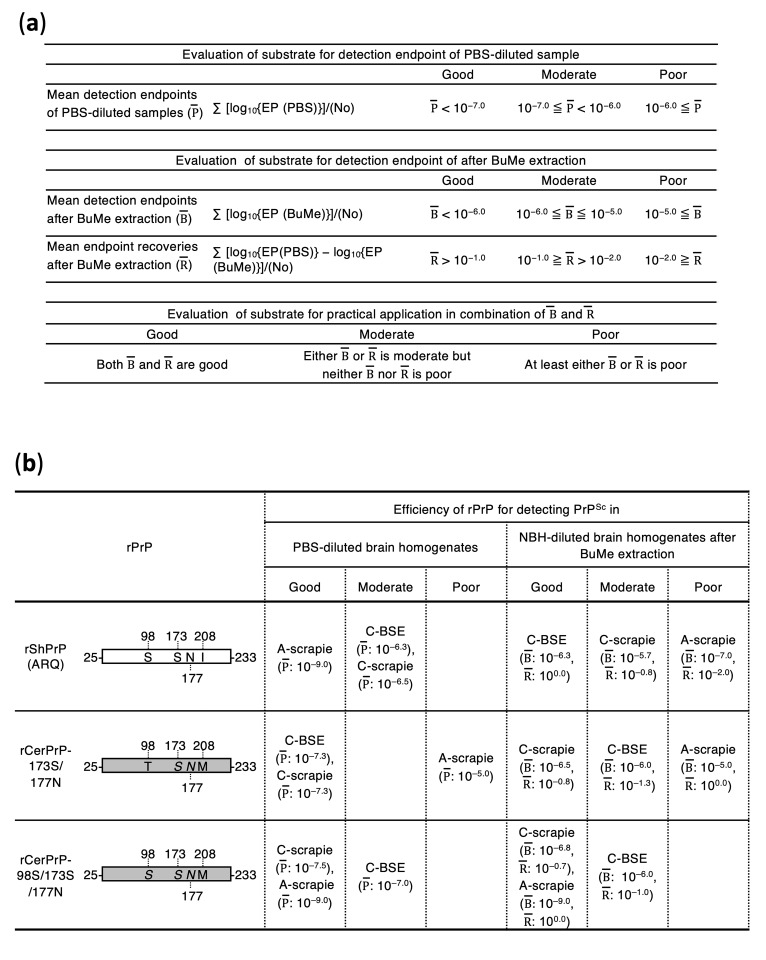
Summary of rPrP reactivity for the detection of C-BSE, C-scrapie, and A-scrapie. (**a**) Criteria used for evaluating rPrP performance in RT-QuIC. The mean detection endpoint of PBS-diluted samples ($\bar{P}$) was used as an indicator of basic detection efficacy and classified into three levels: good, moderate, and poor. Practical performance was evaluated using a combination of the mean detection endpoint after BuMe extraction ($\bar{B}$) and the mean endpoint recovery after BuMe extraction ($\bar{R}$). EP and No indicate endpoint and number of cases, respectively. (**b**) Evaluation of rPrP for detecting C-BSE, C-scrapie, and A-scrapie. rShPrP, rCerPrP-173S/177N, and rCerPrP-98S/173S/177N are indicated with their aa differences. rShPrP and rCerPrP backbones are represented by white and gray bars, respectively. Aa substitutions are indicated using single-letter codes; italic letters represent ShPrP-specific residues introduced by gene engineering, whereas Roman letters indicate authentic residues in each species.

Overall, combining single-step BuMe (3:1) lipid extraction with appropriate rPrP substrate selection enabled the development of suitable conditions for RT-QuIC detection of C-BSE and C- and A-scrapie in practical settings, i.e., low levels of prions in high concentrations of CNS homogenates. Based on these results, rShPrP, rCerPrP-173S/177N or rCerPrP-98S/173S/177N, and rCerPrP-98S/173S/177N are recommended as substrates for RT-QuIC detection of C-BSE, C-scrapie, and A-scrapie, respectively (Figure 3).

### 3.4. Inhibition of RT-QuIC by Major Phospholipids in Brain Tissue

As described above, BuMe (3:1) lipid extraction improved RT-QuIC performance. Although phospholipids in brain tissue have been known to inhibit RT-QuIC assay [20], the specific lipid species responsible has remained unclear. Therefore, we tested the inhibitory effects of the phospholipids, PC, PE, PS, and SM, all mainly present in the cell membrane bilayer, at the physiological concentrations in human brain tissue: 0.002% PC, 0.003% PE; 0.0003% PS; and 0.0013% SM [29]. We also tested each lipid at the total phospholipid concentration (0.007%) as the highest concentration, and at the PS concentration (0.0003%), i.e., as the lowest physiological level (Table 5). The results revealed that SM alone strongly inhibited RT-QuIC at the physiological concentration. Additionally, the reactions were completely inhibited by a mixture of three glycerophospholipids—PC, PE, and PS—at physiological levels despite these phospholipids’ mild individual effects.

## 4. Discussion

The reactivity of rCerPrP to CWD and atypical BSE prions in RT-QuIC was previously shown to be minimally affected by the presence of NBH from the corresponding host species, enabling practical and sensitive RT-QuIC for detecting CWD and atypical BSE prions [25]. In contrast, robust conditions for detecting low levels of C-BSE and scrapie prions in the CNS still require improvements, despite the evaluation of 15 different rPrPs [25]. Because endogenous brain lipids can inhibit RT-QuIC assays and EtOH extraction partially improves assay performance [20], we attempted to develop a practical RT-QuIC for C-BSE and scrapie prion detection by combining lipid removal with rPrP substrate optimization. Our results confirmed that proteins present in brain homogenates, including PrP^C^, were not major inhibitors of RT-QuIC (Figure S3) and that EtOH extraction removed inhibitory factors, as previously reported [20]; however, repeated EtOH extractions were required to achieve sufficient sensitivity (Table 1). Notably, we demonstrated that BuMe (3:1) extraction [33] provided a simple and effective method for removing inhibitory lipids from brain homogenates.

The efficacy of water-soluble alcohol-based lipid extraction depended on the alkyl chain length and the solvent-to-sample ratio [31,32]. Although BuOH dissolved hydrophobic lipids and showed lower water solubility relative to EtOH and MeOH, combining BuOH with MeOH improved solubility and enhanced the extraction of polar and nonpolar lipids [33]. BuMe (3:1) extracted sphingolipids, such as SM, and non-acidic phospholipids, such as PC and PE, with an efficiency comparable to that of the Folch method using chloroform and MeOH [35], and also showed superior extraction of acidic phospholipids, such as PS [33]. In the present study, these phospholipids individually or synergistically inhibited RT-QuIC assays (Table 5); however, BuMe (3:1) effectively removed these inhibitors in a single step. Other possible effects of BuMe (3:1) extraction can be a simultaneous removal of other components present in the brain tissue homogenate such as glycosaminoglycans that can bind with PrP and may affect the RT-QuIC assay as a potential inhibitor [36].

Pretreatment of tissue homogenates with alcohol [20], iron oxide magnetic extraction [23], and silica nanoparticles [37] have been reported to improve the detection sensitivity of CWD prions in RT-QuIC by removing inhibitors, concentrating PrP^Sc^, and/or increasing PrP^Sc^ and rPrP density on nanoparticle surfaces; however, their utility for C-BSE and scrapie prion detection has remained unclear [38,39]. Here, our results demonstrated that single-step BuMe (3:1) extraction effectively removed RT-QuIC inhibitors. Combined with this pretreatment, we evaluated rPrP substrates for detecting C-BSE and C- and A-scrapie using two primary criteria: (i) a detection endpoint after BuMe extraction and (ii) an endpoint recovery after BuMe extraction relative to the PBS-diluted samples (Figure 3a). Additionally, lag-phase prolongation after BuMe extraction was considered. Based on these measures, the overall rPrP performance in RT-QuIC was evaluated (Figure 3).

In the current study, three rPrPs, namely rCerPrP-173S/177N, rCerPrP-98S/173S/177N, and rShPrP, were evaluated. Following BuMe extraction, the detection of C-BSE cases using rShPrP recovered to levels comparable to those of PBS-diluted samples (mean endpoint [$\bar{B}$] and recovery after BuMe extraction [$\bar{R}$]: 10^−6.3^ and 10^0.0^, respectively; Figure 3b). In contrast, the endpoint recovery after the BuMe extraction for C- and A-scrapie prions was less efficient ($\bar{B}\;and\;\bar{R}\;$ for C-scrapie cases: 10^−5.7^ and 10^−0.8^, respectively; $\bar{B}\;and\;\bar{R}\;$ for A-scrapie: 10^−7.0^ and 10^−2.0^, respectively, Figure 3b), indicating that rShPrP was the most suitable substrate for C-BSE detection (Figure 3b). The remaining two rPrP substrates showed moderate performance for C-BSE detection, where $\bar{B}\;and\;\bar{R}$ were 10^−6.0^ and 10^−1.3^ for rCerPrP-173S/177N, respectively, and 10^−6.0^ and 10^−1.0^ for rCerPrP-98S/173S/177N, respectively (Figure 3b). For C-scrapie, rCerPrP-173S/177N exhibited suitable performance ($\bar{B}\;and\;\bar{R}$: 10^−6.5^ and 10^−0.8^, respectively), and rCerPrP-98S/173S/177N showed comparable results ($\bar{B}\;and\;\bar{R}$: 10^−6.8^ and 10^−0.7^, respectively; Figure 3b), supporting their practical utility (Figure 3b). In contrast, the reactivities of these rPrP substrates to A-scrapie differed markedly, as rCerPrP-98S/173S/177N exhibited a desirable performance ($\bar{B}\;and\;\bar{R}$: 10^−9.0^ and 10^0.0^, respectively), while rCerPrP-173S/177N was ineffective owing to an insufficient detection sensitivity ($\bar{B}\;and\;\bar{R}$: 10^−5.0^ and 10^0^, respectively; Figure 3b), despite only one amino acid difference at codon 98. Although 1–2 log reductions in the detection endpoint were observed after BuMe extraction depending on the combination of seeds and substrates (Figure 2 and Table 2, Table 3 and Table 4), the use of appropriate substrates—such as rShPrP for C-BSE, rCerPrP-173S/177N or rCerPrP-98S/173S/177N for C-scrapie, and rCerPrP-98S/173S/177N for A-scrapie—resulted in better endpoint recoveries (>10^−1.0^), corresponding to less than 1-log reduction in the detection endpoint.

To date, 14 rPrPs, including nine full-length rPrPs (Bv [M_109_ and I_109_], Bo, Ha, human [Hu], Mo, and Sh [A_136_R_154_Q_171_, V_136_R_154_Q_171_, and A_136_R_154_R_171_]) [40,41,42,43,44,45], N-terminally truncated rBvPrP [42] and rHaPrP [42,43], E211K mutant of rBoPrP [40], and chimeric Hu-Bv rPrP [42] and Ha-Sh rPrP [42,43], have been tested for detecting C-BSE, C-scrapie, and A-scrapie. Full-length rBvPrP (M_109_) and Ha-Sh rPrP detected PBS-diluted C-BSE prions up to 10^−5^ and 10^−6^ [42,43], respectively. Additionally, rBvPrP, Ha-S rPrP, and rShPrP detected PBS-diluted C-scrapie up to 10^−6^, 10^−8^, and 10^−7^, respectively, within 40 h [18,44,46], and the detection endpoint of A-scrapie using rBvPrP (M_109_) reached 10^−7^ [44]. Nearly all previous studies used PBS-diluted samples [18,40,41,42,43,44,46], leaving the detection of low levels of prions in high concentrations of CNS homogenates largely unexplored. In the present study, our RT-QuIC assay detected C-BSE prions using rShPrP, C-scrapie prions using rCerPrP-173S/177N or rCerPrP-98S/173S/177N, and A-scrapie prions using rCerPrP-98S/173S/177N up to $\bar{B}$ at 10^–6.3^, 10^−6.5^ or 10^−6.8^, and 10^−9.0^, respectively (Figure 3b). Although $\bar{B}$ values for C-BSE and C-scrapie may be modest, they were comparable with previously reported RT-QuIC sensitivities for C-BSE (10^−6^) [43] and those for C-scrapie (10^−7^–10^−8^) [18,44,46]. Despite inclusion of only one A-scrapie case, the detection endpoint exceeded that which was previously reported (10^−7^) [44], even seeds were present in high concentrations of brain homogenates. These findings indicated that combining single-step BuMe (3:1) lipid extraction with an appropriate rPrP selection overcame the key limitations of RT-QuIC.

RT-QuIC can detect amyloid seeding activity in the diluted seeds containing 1 to 10 femtograms (fg) of PrP-res equivalent from sheep and deer [22]. The detection limit of PBS-diluted C-scrapie Y5 reached 10^–9^ dilution. Based on the quantification of PrP-res in the brain homogenates (Table S1), the final reaction mixture at this dilution contained approximately 30 fg of PrP-res per well. Thus, the detection limit of RT-QuIC for C-scrapie in this study was estimated as several tens of femtograms, which was nearly comparable to the previous report [22]. For C-BSE WA, the detection limit of PBS-diluted sample reached 10^−8^ dilution. Although the relationship between RT-QuIC detection limit for C-BSE and the amount of PrP-res has not been thoroughly investigated, the highest dilution of brain homogenates yielding a positive reaction was similar to that which was previously reported [42], suggesting that the detection limit for C-BSE in this study was likewise comparable to earlier findings.

A single amino acid difference at codon 98 between rCerPrP-173S/177N and rCerPrP-98S/173S/177N in the N-terminus markedly altered A-scrapie detection, with the former showing poor reactivity even in PBS-diluted samples. These results indicated that Ser98 in the N-terminal region was important for the reactivity of rCerPrP-98S/173S/177N to A-scrapie prions. Indeed, there is a line of evidence that has demonstrated the influence of the N-terminal region around the amino acid position at 98 on the conformational conversion of PrP^C^. *Prnp*^0/0^ mice expressing PrP with a deletion of amino acids 91–106 were resistant to RML, 22L, and FK-1 strains but susceptible to mouse-adapted BSE strain with prolonged incubation periods, suggesting that the influence of this region on PrP^C^ and on the PrP^Sc^ formation was strain dependent [47,48]. A Ser to Thr substitution at codon 98 in ShPrP reduced the conversion efficiency of rShPrP in PMCA seeded with in vitro amplified scrapie prions but improved that of rShPrP seeded with in vitro amplified CWD prions [34]. These findings supported the idea that the single amino acid difference at codon Thr/Ser98 in the rCerPrP backbone can result in a different reactivity to A-scrapie prions.

Similarly, the difference at codon 208 between rShPrP and rCerPrP-98S/173S/177N affected their reactivity to each prion, especially to A-scrapie as the reaction of rShPrP was severely inhibited by NBH. These results suggested that there was the significant involvement of amino acid residue at position 208, located in hydrophobic interface between α1- and α3-helices [34,49,50], on the reactivity to each prion in RT-QuIC. Indeed, substituting Ile208 in rShPrP with Met reduced its conformational conversion with in vitro amplified scrapie prions but enhanced the conversion with in vitro amplified CWD prions [34]. Also, the substitution of Met205 in BvPrP, which corresponds to Ile/Met208 in ShPrP/CerPrP, to Ile in rabbit PrP inhibited the conformational conversion of rBvPrP with CWD prions [51]. These results suggested that the amino acid at position 208 was one of the important amino acids modulating the conformational conversion of rPrP. The differential reactivities of rCerPrP-173S/177N and rCerPrP-98S/173S/177N or rShPrP and rCerPrP-98S/173S/177N toward C- and A-scrapie prions may facilitate discrimination between these two scrapie prions, similar to approaches using multiple rPrPs in RT-QuIC to distinguish C-BSE from atypical BSE [42,43,45].

Naturally occurring animal prion disease cases may differ in PrP^Sc^ levels and amyloid seeding activity due to differences in the disease stage and strain properties. The C-BSE and C-scrapie samples used in the present study were collected in the 2000s and 1990s, respectively; however, because knowledge of the appropriate brain regions to sample was limited at that time, the collected brain regions were not exactly the same across these cases. In addition, long-term storage may have affected sample quality. These factors have the potential to influence RT-QuIC outcomes. Moreover, given that the limited BSE and scrapie cases were used in the present study, further examination using larger number of samples collected from a wider geographical area will be required to generalize methods for practical detection of diverse strains and/or disease cases of C-BSE, and C- and A-scrapie, including samples with different PrP^Sc^ levels.

## 5. Conclusions

This study aimed to establish practical RT-QuIC for detecting C-BSE and C- and A-scrapie. A single-step lipid extraction using a 3:1 mixture of 2-butanol and methanol was effective as a pretreatment to remove inhibitors from brain homogenates. Among the three rPrPs extensively evaluated, rShPrP was the most suitable substrate for the practical detection of C-BSE prions. rCerPrP-173S/177N and rCerPrP-98S/173S/177N showed desirable performance for detecting C-scrapie prions. Moreover, rCerPrP-98S/173S/177N, but not rCerPrP-173S/177N, was identified as an optimal substrate for detecting A-scrapie prions. Our results have emphasized that single-step BuMe (3:1) lipid extraction substantially and practically improves RT-QuIC, highlighting the importance of rPrP substrate selection for optimizing RT-QuIC assays. However, identifying broader applicable conditions for diverse prion strains and samples remains an important direction for further expanding RT-QuIC versatility.

## Figures and Tables

**Table 1:** Effect of single-step lipid removal by ethanol and butanol/methanol.

Seed	Diluent/ Extraction	Lag Phase (h) ^6^ (Positive Wells/Total Wells)						End-Point
		10^−4^	10^−5^	10^−6^	10^−7^	10^−8^	10^−9^	
22L	PBS ^1^	13.5 ± 5.4 (9/9)	23.8 ± 6.6 (9/9)	28.3 ± 5.6 (8/9)	34.9 ± 11.3 (8/9)	47.6 ± 9.0 (6/9)	46.0 ± 10.5 (5/6)	<10^−9^
	NBH ^2^	34.0 (1/9)	52.8 (1/9)	>60.0 (0/9)	>60.0 (0/9)	>60.0 (0/9)	>60.0 (0/9)	10^−5^
	NBH/ EtOH × 1 ^3^	34.7 ± 11.9 ** (9/9)	36.2 ± 11.3 * (8/9)	43.8 ± 9.9 ** (7/9)	>60.0 (0/9)	>60.0 (0/9)	52.7 (1/9)	10^−6^
	NBH/ EtOH × 2 ^4^	28.8 ± 9.1 ** (9/9)	38.6 ± 6.4 ** (9/9)	48.0 ± 9.2 ** (7/9)	49.0 ± 9.1 (5/9)	>60.0 (0/9)	>60.0 (0/9)	10^−7^
	NBH/ BuMe (3:1) ^5^	26.3 ± 6.9 ** (9/9)	31.2 ± 7.6 * (9/9)	39.6 ± 7.5 * (8/9)	40.6 ± 9.4 (7/9)	54.0 (1/9)	59.3 (1/9)	<10^−9^

^1^ Seeds (22L prion-infected brain homogenates) were diluted with PBS. ^2^ Seeds were diluted with 2% mouse NBH in PBS. ^3^ Seeds diluted with 2% mouse NBH were extracted with EtOH once. ^4^ Seeds diluted with 2% mouse NBH were extracted with EtOH twice. ^5^ Seeds diluted with 2% mouse NBH were extracted with BuMe (3:1) once. ^6^ Mean ± SD were calculated using wells that were determined to be positive out of the total numbers of wells used in three independent experiments with triplicate wells. Asterisks indicate that lag phase(s) after treatment was significantly prolonged compared to the corresponding dilution of PBS-diluted seed. The statistical analyses were performed by Mann-Whiteney’s *U*-test for non-normal distribution or by *F*-test to assess variance levels, followed by a Welch’s *t*-test for normal distribution. *: *p* < 0.05, **: *p* < 0.01.

**Table 2:** Effect of single-step lipid removal by butanol/methanol for the detection of C-BSE.

Seed	rPrP	Diluent ^ 1^	Extraction ^2^	Lag Phase (h) ^3^ (Positive Wells/Total Wells)						End-Point
				10^−4^	10^−5^	10^−6^	10^−7^	10^−8^	10^−9^	
WA	CerPrP-173S/177N	PBS	−	18.6 ± 4.8 (6/9)	26.3 ± 5.2 (9/9)	37.9 ± 9.2 (9/9)	47.4 ± 16.3 (3/9)	51.2 (1/9)	>55.0 (0/9)	10^−8^
		Bo NBH	−	>55.0 (0/9)	>55.0 (0/9)	>55.0 (0/9)	>55.0 (0/9)	>55.0 (0/9)	>55.0 (0/9)	>10^−4^
			+	26.2 ± 3.6 * (9/9)	34.6 ± 6.4 * (9/9)	41.4 ± 5.8 (7/9)	>55.0 (0/9)	>55.0 (0/9)	>55.0 (0/9)	10^−6^
	CerPrP-98S/ 173S/177N	PBS	−	>55.0 (0/9)	25.9 ± 3.5 (9/9)	33.6 ± 7.7 (9/9)	34.8, 50.5 (2/9)	38.7, 43.8 (2/9)	>55.0 (0/9)	10^−8^
		Bo NBH	−	>55.0 (0/9)	>55.0 (0/9)	>55.0 (0/9)	>55.0 (0/9)	>55.0 (0/9)	>55.0 (0/9)	>10^−4^
			+	25.8 ± 3.8 (9/9)	41.8 ± 8.5 ** (8/9)	43.1 ± 9.5 * (4/9)	>55.0 (0/9)	>55.0 (0/9)	>55.0 (0/9)	10^−6^
	ShPrP (ARQ)	PBS	−	30.7 ± 5.9 (9/9)	32.9 ± 3.0 (9/9)	45.7 ± 8.0 (5/9)	34.8 (1/9)	54.0 (1/9)	>55.0 (0/9)	10^−8^
		Bo NBH	−	>55.0 (0/9)	>55.0 (0/9)	>55.0 (0/9)	>55.0 (0/9)	>55.0 (0/9)	>55.0 (0/9)	>10^−4^
			+	29.7 ± 5.2 (9/9)	39.3 ± 8.3 (9/9)	44.6 ± 10.2 (3/9)	47.2 (1/9)	>55.0 (0/9)	>55.0 (0/9)	10^−7^
KUS	CerPrP-173S/177N	PBS	−	>55.0 (0/3)	19.9 ± 1.5 (3/3)	27.5 ± 2.0 (3/3)	42.2 (1/3)	>55.0 (0/3)	>55.0 (0/3)	10^−7^
		Bo NBH	−	>55.0 (0/3)	>55.0 (0/3)	>55.0 (0/3)	>55.0 (0/3)	>55.0 (0/3)	>55.0 (0/3)	>10^−4^
			+	29.8 ± 2.3 (3/3)	32.7 ± 4.5 * (3/3)	42.3 ± 4.9 * (3/3)	>55.0 (0/3)	>55.0 (0/3)	>55.0 (0/3)	10^−6^
	CerPrP-98S/ 173S/177N	PBS	−	35.0, 43.2 (2/3)	17.5 ± 2.3 (3/3)	26.6 ± 11.3 (3/3)	45.2 (1/3)	51.8 (1/3)	>55.0 (0/3)	10^−8^
		Bo NBH	−	44.0 (1/3)	>55.0 (0/3)	>55.0 (0/3)	>55.0 (0/3)	>55.0 (0/3)	>55.0 (0/3)	10^−4^
			+	24.2 ± 0.7 (3/3)	28.5 ± 3.1 * (3/3)	38.6 ± 6.9 (3/3)	39.7, 52.8 (2/3)	>55.0 (0/3)	>55.0 (0/3)	10^−7^
	ShPrP (ARQ)	PBS	−	21.3 ± 4.2 (3/3)	30.4 ± 2.5 (3/3)	39.2 ± 7.4 (3/3)	>55.0 (0/3)	>55.0 (0/3)	>55.0 (0/3)	10^−6^
		Bo NBH	−	32.7, 54.2 (2/3)	>55.0 (0/3)	>55.0 (0/3)	>55.0 (0/3)	>55.0 (0/3)	>55.0 (0/3)	10^−4^
			+	27.2 ± 10.2 (3/3)	38.0 ± 8.6 (3/3)	43.3, 48.7 (2/3)	>55.0 (0/3)	>55.0 (0/3)	>55.0 (0/3)	10^−6^
TE	CerPrP-173S/177N	PBS	−	51.2 ± 4.2 (3/3)	25.1 ± 2.9 (3/3)	31.2, 39.7 (2/3)	27.5 (1/3)	>55.0 (0/3)	>55.0 (0/3)	10^−7^
		Bo NBH	−	>55.0 (0/3)	>55.0 (0/3)	>55.0 (0/3)	>55.0 (0/3)	>55.0 (0/3)	>55.0 (0/3)	>10^−4^
			+	27.6 ± 1.1 (3/3)	53.5 ± 3.3 * (3/3)	44.7 (1/3)	>55.0 (0/3)	>55.0 (0/3)	>55.0 (0/3)	10^−6^
	CerPrP-98S/ 173S/177N	PBS	−	40.3, 43.8 (2/3)	30.3 ± 4.1 (3/3)	50.8 (1/3)	>55.0 (0/3)	>55.0 (0/3)	>55.0 (0/3)	10^−6^
		Bo NBH	−	>55.0 (0/3)	>55.0 (0/3)	>55.0 (0/3)	>55.0 (0/3)	>55.0 (0/3)	>55.0 (0/3)	>10^−4^
			+	28.4 ± 5.0 (3/3)	35.9 ± 3.4 (3/3)	>55.0 (0/3)	>55.0 (0/3)	>55.0 (0/3)	>55.0 (0/3)	10^−5^
	ShPrP (ARQ)	PBS	−	29.9 ± 1.4 (3/3)	43.1 ± 10.4 (3/3)	>55.0 (0/3)	>55.0 (0/3)	>55.0 (0/3)	>55.0 (0/3)	10^−5^
		Bo NBH	−	>55.0 (0/3)	>55.0 (0/3)	>55.0 (0/3)	>55.0 (0/3)	>55.0 (0/3)	>55.0 (0/3)	>10^−4^
			+	25.5 ± 2.4 (3/3)	34.3 ± 8.2 (3/3)	46.8 (1/3)	>55.0 (0/3)	>55.0 (0/3)	>55.0 (0/3)	10^−6^

^1^ C-BSE-affected cattle brains were diluted with PBS or 2% bovine (Bo) NBH at 10^−4^–10^−9^ dilutions. ^2^ + and − indicate with or without lipid extraction using BuMe (3:1). ^3^ Mean ± SD were calculated using wells that were determined to be positive out of the total numbers of wells used in three independent experiments or one experiment with triplicate wells. Asterisks indicate that lag phase(s) of NBH diluted samples before or after BuMe extraction was significantly prolonged compared to the corresponding dilution of PBS-diluted seed. Statistical analyses were performed by Mann-Whiteney’s *U*-test for non-normal distribution or by *F*-test to assess variance levels, followed by Welch’s *t*-test for normal distribution *: *p* < 0.05, **: *p* < 0.01.

**Table 3:** Effect of single-step lipid removal by butanol/methanol for the detection of C-scrapie prions.

Seed	rPrP	Diluent ^ 1^	Extraction ^2^	Lag Phase (h) ^3^ (Positive Wells/Total Wells)						End-Point
				10^−4^	10^−5^	10^−6^	10^−7^	10^−8^	10^−9^	
Y5	CerPrP-173S/177N	PBS	−	10.2 ± 1.9 (9/9)	12.8 ± 3.4 (9/9)	19.2 ± 5.3 (9/9)	29.2 ± 9.0 (9/9)	42.9 ± 5.4 (2/9)	40.5 (1/9)	<10^−9^
		Sh NBH	−	31.3 ± 9.1 ** (9/9)	41.7 ± 3.3 ** (4/9)	42.8 (1/9)	51.0 (1/9)	>55.0 (0/9)	>55.0 (0/9)	10^−7^
			+	18.4 ± 4.2 ** (9/9)	29.2 ± 6.9 ** (9/9)	40.4 ± 5.5 ** (9/9)	43.3, 51.8 (2/9)	44.8, 46.2 (2/9)	>55.0 (0/9)	10^−8^
	CerPrP-98S/ 173S/177N	PBS	−	15.7 ± 14.1 (9/9)	11.1 ± 1.8 (9/9)	15.5 ± 2.7 (9/9)	25.6 ± 4.4 (9/9)	43.8 ± 7.3 (5/9)	33.3, 46.3 (2/9)	<10^−9^
		Sh NBH	−	42.2 ± 8.3 * (4/9)	>55.0 (0/9)	>55.0 (0/9)	>55.0 (0/9)	>55.0 (0/9)	>55.0 (0/9)	10^−4^
			+	17.4 ± 3.9 * (9/9)	28.4 ± 6.8 ** (9/9)	38.5 ± 8.0 ** (9/9)	44.7 ± 9.6 ** (3/9)	44.3, 48.7 (2/9)	>55.0 (0/9)	10^−8^
	ShPrP (ARQ)	PBS	−	16.9 ± 1.4 (9/9)	16.2 ± 4.4 (9/9)	30.2 ± 14.6 (8/9)	50.0 ± 4.7 (3/9)	>55.0 (0/9)	>55.0 (0/9)	10^−7^
		Sh NBH	−	33.8 ± 14.3 ** (8/9)	37.5 ± 10.2 ** (8/9)	45.0 ± 11.3 (3/9)	41.2 (1/9)	>55.0 (0/9)	>55.0 (0/9)	10^−7^
			+	41.1 ± 9.7 ** (9/9)	42.6 ± 6.8 ** (8/9)	49.3 ± 1.9 (3/9)	>55.0 (0/9)	>55.0 (0/9)	>55.0 (0/9)	10^−6^
B3	CerPrP-173S/177N	PBS	−	29.4 ± 3.4 (3/3)	27.3 ± 3.2 (3/3)	44.1 ± 6.5 (3/3)	>55.0 (0/3)	>55.0 (0/3)	>55.0 (0/3)	10^−6^
		Sh NBH	−	>55.0 (0/3)	>55.0 (0/3)	>55.0 (0/3)	>55.0 (0/3)	>55.0 (0/3)	>55.0 (0/3)	>10^−4^
			+	34.8 ± 6.1 (3/3)	47.3 ± 2.3 * (3/3)	49.3 (1/3)	>55.0 (0/3)	>55.0 (0/3)	>55.0 (0/3)	10^−6^
	CerPrP-98S/ 173S/177N	PBS	−	25.5 ± 4.4 (3/3)	23.4 ± 3.9 (3/3)	51.8 (1/3)	37.2 (1/3)	>55.0 (0/3)	>55.0 (0/3)	10^−7^
		Sh NBH	−	>55.0 (0/3)	>55.0 (0/3)	>55.0 (0/3)	>55.0 (0/3)	>55.0 (0/3)	>55.0 (0/3)	>10^−4^
			+	31.1 ± 2.0 * (3/3)	36.5 ± 4.0 * (3/3)	52.7 (1/3)	>55.0 (1/3)	>55.0 (0/9)	>55.0 (0/9)	10^−7^
	ShPrP (ARQ)	PBS	−	15.6 ± 3.4 (3/3)	35.8 ± 5.6 (3/3)	42.3 ± 7.4 (3/3)	>55.0 (0/3)	>55.0 (0/3)	>55.0 (0/3)	10^−6^
		Sh NBH	−	25.8, 35.7 (2/3)	52.8 (1/3)	>55.0 (0/3)	>55.0 (0/3)	>55.0 (0/3)	>55.0 (0/3)	10^−5^
			+	28.5 ± 1.8 * (3/3)	37.8 ± 6.9 (3/3)	42.1 ± 10.8 (3/3)	>55.0 (0/3)	>55.0 (0/3)	>55.0 (0/3)	10^−6^
G1	CerPrP-173S/177N	PBS	−	24.0, 31.3 (2/3)	19.9 ± 1.5 (3/3)	27.0 ± 2.8 (3/3)	42.3 (1/3)	>55.0 (0/3)	>55.0 (0/3)	10^−7^
		Sh NBH	−	>55.0 (0/3)	>55.0 (0/3)	>55.0 (0/3)	>55.0 (0/3)	>55.0 (0/3)	>55.0 (0/3)	>10^−4^
			+	24.8 ± 0.6 (3/3)	36.2 ± 13.3 (3/3)	41.7 (1/3)	>55.0 (0/3)	>55.0 (0/3)	>55.0 (0/3)	10^−6^
	CerPrP-98S/ 173S/177N	PBS	−	23.7 ± 1.8 (3/3)	18.2 ± 1.9 (3/3)	31.1 ± 3.2 (3/3)	>55.0 (0/3)	>55.0 (0/3)	>55.0 (0/3)	10^−6^
		Sh NBH	−	>55.0 (0/3)	>55.0 (0/3)	>55.0 (0/3)	>55.0 (0/3)	>55.0 (0/3)	>55.0 (0/3)	>10^−4^
			+	29.3 ± 3.8 * (3/3)	37.2 ± 3.1 * (3/3)	39.2, 48.3 (2/3)	>55.0 (0/3)	>55.0 (0/3)	>55.0 (0/3)	10^−6^
	ShPrP (ARQ)	PBS	−	23.5 ± 8.6 (3/3)	24.0, 37.7 (2/3)	>55.0 (0/3)	>55.0 (0/3)	>55.0 (0/3)	>55.0 (0/3)	10^−5^
		Sh NBH	−	44.1 ± 9.1 (3/3)	>55.0 (0/3)	>55.0 (0/3)	>55.0 (0/3)	>55.0 (0/3)	>55.0 (0/3)	10^−4^
			+	42.9 ± 4.1 * (3/3)	44.3, 47.3 (2/3)	>55.0 (0/3)	>55.0 (0/3)	>55.0 (0/3)	>55.0 (0/3)	10^−5^
KH2	CerPrP-173S/177N	PBS	−	11.5 ± 3.7 (3/3)	9.2 ± 1.6 (3/3)	11.8 ± 2.2 (3/3)	17.9 ± 1.7 (3/3)	26.8 (1/3)	>55.0 (0/3)	10^−8^
		Sh NBH	−	33.4 ± 2.4 ** (3/3)	29.2 ± 5.7 ** (3/3)	35.7, 42.7 (2/3)	51.5 (1/3)	>55.0 (0/3)	>55.0 (0/3)	10^−7^
			+	14.7 ± 0.2 ** (3/3)	16.6 ± 1.8 * (3/3)	24.9 ± 1.0 * (3/3)	29.0 (1/3)	>55.0 (0/3)	>55.0 (0/3)	10^−7^
	CerPrP-98S/ 173S/177N	PBS	−	9.5 ± 1.5 (3/3)	8.4 ± 1.3 (3/3)	10.9 ± 0.9 (3/3)	20.5 ± 6.6 (3/3)	31.3 (1/3)	>55.0 (0/3)	10^−8^
		Sh NBH	−	32.8 ± 7.0 * (3/3)	27.5, 32.8 (2/3)	37.3, 37.5 (2/3)	>55.0 (0/3)	>55.0 (0/3)	>55.0 (0/3)	10^−6^
			+	11.1 ± 0.9 (3/3)	11.7 ± 1.1 * (3/3)	33.1 ± 3.1 ** (3/3)	34.8, 35.7 (2/3)	>55.0 (0/9)	>55.0 (0/3)	10^−7^
	ShPrP (ARQ)	PBS	−	11.2 ± 3.5 (3/3)	10.6 ± 0.3 (3/3)	16.3 ± 4.0 (3/3)	16.9 ± 3.0 (3/3)	>55.0 (0/3)	>55.0 (0/3)	10^−7^
		Sh NBH	−	17.8 ± 1.0 (3/3)	23.1 ± 3.8 * (3/3)	28.2 ± 5.9 (3/3)	36.8 ± 8.4 * (3/3)	>55.0 (0/3)	>55.0 (0/3)	10^−7^
			+	16.1 ± 2.0 (3/3)	19.0 ± 3.1 (3/3)	25.4 ± 8.3 (2/3)	>55.0 (0/3)	>55.0 (0/3)	>55.0 (0/3)	10^−6^
S2	CerPrP-173S/177N	PBS	−	24.9 ± 5.0 (3/3)	26.3 ± 3.4 (3/3)	24.3 ± 5.2 (3/3)	55.3 (1/3)	>55.0 (0/3)	>55.0 (0/3)	10^−7^
		Sh NBH	−	>55.0 (0/3)	>55.0 (0/3)	>55.0 (0/3)	>55.0 (0/3)	>55.0 (0/3)	>55.0 (0/3)	>10^−4^
			+	31.4 ± 2.3 (3/3)	46.3, 49.3 (2/3)	48.5, 52.2 (2/3)	>55.0 (0/3)	>55.0 (0/3)	>55.0 (0/3)	10^−6^
	CerPrP-98S/ 173S/177N	PBS	−	27.3 ± 3.8 (3/3)	22.1 ± 0.7 (3/3)	26.9 ± 1.9 (3/3)	42.0, 47.3 (2/3)	>55.0 (0/3)	>55.0 (0/3)	10^−7^
		Sh NBH	−	>55.0 (0/3)	>55.0 (0/3)	>55.0 (0/3)	>55.0 (0/3)	>55.0 (0/3)	>55.0 (0/3)	>10^−4^
			+	30.1 ± 4.5 (3/3)	44.0 ± 9.7 (3/3)	34.0, 48.8 (2/3)	>55.0 (0/3)	34.0 (1/3)	>55.0 (0/3)	10^−6^
	ShPrP (ARQ)	PBS	−	20.1 ± 1.6 (3/3)	24.4 ± 7.2 (3/3)	42.5 ± 6.1 (3/3)	44.0, 47.3 (2/3)	>55.0 (0/3)	>55.0 (0/3)	10^−7^
		Sh NBH	−	35.8 ± 5.1 (3/3)	40.5, 50.2 (2/3)	>55.0 (0/3)	>55.0 (0/3)	>55.0 (0/3)	>55.0 (0/3)	10^−5^
			+	36.4 ± 7.1 ** (3/3)	45.8 ± 4.6 * (3/3)	>55.0 (0/3)	>55.0 (0/3)	>55.0 (0/3)	>55.0 (0/3)	10^−5^
S3	CerPrP-173S/177N	PBS	−	36.4 ± 8.1 (3/3)	20.4 ± 3.0 (3/3)	28.4 ± 3.5 (3/3)	43.3, 51.0 (2/3)	>55.0 (0/3)	>55.0 (0/3)	10^−7^
		Sh NBH	−	>55.0 (0/3)	>55.0 (0/3)	>55.0 (0/3)	>55.0 (0/3)	>55.0 (0/3)	>55.0 (0/3)	>10^−4^
			+	25.1 ± 6.6 (3/3)	38.5 ± 7.7 * (3/3)	24.8, 54.2 (2/3)	>55.0 (0/3)	>55.0 (0/3)	>55.0 (0/3)	10^−6^
	CerPrP-98S/ 173S/177N	PBS	−	28.8 ± 7.8 (3/3)	21.1 ± 3.7 (3/3)	29.3 ± 1.9 (3/3)	40.7 (1/3)	52.3 (1/3)	>55.0 (0/3)	10^−8^
		Sh NBH	−	>55.0 (0/3)	>55.0 (0/3)	>55.0 (0/3)	>55.0 (0/3)	>55.0 (0/3)	>55.0 (0/3)	10^−4^
			+	30.3 ± 2.4 (3/3)	29.8 ± 2.4 (3/3)	44.2 ± 4.8 (3/3)	53.3 (1/3)	>55.0 (0/3)	>55.0 (0/3)	10^−7^
	ShPrP (ARQ)	PBS	−	18.0 ± 2.4 (3/3)	19.1 ± 8.4 (3/3)	35.8 ± 5.9 (3/3)	29.8, 48.7 (2/3)	>55.0 (0/3)	>55.0 (0/3)	10^−7^
		Sh NBH	−	39.2, 45.0 (2/3)	43.0 (1/3)	>55.0 (0/3)	>55.0 (0/3)	>55.0 (0/3)	>55.0 (0/3)	10^−5^
			+	41.9 ± 13.4 * (3/3)	31.0 ± 8.4 (3/3)	38.3, 54.3 (2/3)	>55.0 (0/3)	>55.0 (0/3)	>55.0 (0/3)	10^−6^

^1^ C-scrapie-affected sheep brains were diluted with PBS or 2% sheep (Sh) NBH at 10^−4^–10^−9^ dilutions. ^2,3^ Same as in the footnote of Table 2. *: *p* < 0.05, **: *p* < 0.01.

**Table 4:** Effect of single-step lipid removal by butanol/methanol for the detection of A-scrapie prions.

Seed	rPrP	Diluent ^ 1^	Extraction ^2^	Lag Phase (h) ^3^ (Positive Wells/Total Wells)						End-Point
				10^−4^	10^−5^	10^−6^	10^−7^	10^−8^	10^−9^	
A-scrapie	CerPrP-173S/177N	PBS	−	47.3 ± 4.7 (6/9)	46.4 ± 4.1 (9/9)	>55.0 (0/9)	>55.0 (0/9)	>55.0 (0/9)	>55.0 (0/9)	10^−5^
		Sh NBH	−	>55.0 (0/9)	>55.0 (0/9)	>55.0 (0/9)	>55.0 (0/9)	>55.0 (0/9)	>55.0 (0/9)	>10^−4^
			+	>55.0 (0/9)	>55.0 (0/9)	>55.0 (0/9)	>55.0 (0/9)	>55.0 (0/9)	>55.0 (0/9)	>10^−4^
	CerPrP-98S/ 173S/177N	PBS	−	30.4 ± 3.7 (9/9)	24.5 ± 2.9 (9/9)	48.8 ± 4.0 (6/9)	45.9 ± 7.8 (4/9)	42.9 ± 7.0 (4/9)	53.3 (1/9)	<10^−9^
		Sh NBH	−	46.4 ± 5.3 ** (7/9)	44.9 ± 6.8 ** (8/9)	46.9 ± 4.0 (5/9)	46.2 ± 6.2 (5/9)	48.9 ± 5.0 (5/9)	>55.0 (0/9)	10^−8^
			+	34.3 ± 4.0 * (9/9)	36.3 ± 3.9 ** (9/9)	36.1 ± 3.7 (9/9)	38.7 ± 6.1 (9/9)	40.1 ± 5.8 (7/9)	48.3 ± 5.8 (3/9)	<10^−9^
	ShPrP (ARQ)	PBS	−	32.2 ± 6.8 (9/9)	33.8 ± 10.8 (8/9)	43.1 ± 3.0 (4/9)	44.3, 49.0 (2/9)	35.3, 50.2 (2/9)	38.7, 49.5 (2/9)	<10^−9^
		Sh NBH	−	>55.0 (0/9)	54.2 (1/9)	>55.0 (0/9)	>55.0 (0/9)	52.2 (1/9)	>55.0 (0/9)	10^−5^
			+	47.7 (1/9)	51.0 (1/9)	44.2 (1/9)	49.0, 53.2 (2/9)	>55.0 (0/9)	>55.0 (0/9)	10^−7^

^1^ A-scrapie-affected sheep brain was diluted with PBS or 2% Sh NBH at 10^−4^–10^−9^ dilutions. ^2,3^ Same as in the footnote of Table 2. *: *p* < 0.05, **: *p* < 0.01.

**Table 5:** Inhibitory effects of phospholipids on RT-QuIC reactions.

Seed	Diluents ^1^/Final Concentrations ^2^		Lag Phase (h) ^3^ (Positive Wells/Total Wells)						End-Point
			10^−4^	10^−5^	10^−6^	10^−7^	10^−8^	10^−9^	
22L	PBS	−	13.5 ± 5.4 (9/9)	23.8 ± 6.6 (9/9)	28.3 ± 5.6 (8/9)	34.9 ± 11.3 (8/9)	47.6 ± 9.0 (6/9)	46.0 ± 10.5 (5/6)	<10^−9^
	NBH	0.1%	34.0 (1/9)	52.8 (1/9)	>60.0 (0/9)	>60.0 (0/9)	>60.0 (0/9)	>60.0 (0/9)	10^−5^
	PC	0.007%	>60.0 (0/9)	>60.0 (0/9)	>60.0 (0/9)	>60.0 (0/9)	>60.0 (0/9)	>60.0 (0/9)	>10^−4^
		0.002%	37.6 ± 3.8 ** (9/9)	24.7 ± 15.0 (3/9)	19.8, 23.7 (2/9)	38.3 ± 12.0 * (3/9)	>60.0 (0/9)	>60.0 (0/9)	10^−7^
		0.0003%	9.7 ± 1.9 (9/9)	19.8 ± 6.8 (9/9)	30.1 ± 8.8 (9/9)	33.7 ± 7.8 (7/9)	38.9 ± 9.5 * (8/9)	48.1 ± 13.6 (6/9)	<10^−9^
	PE	(0.007%)	>60.0 (0/9)	32.7 ± 20.9 (3/4)	27.3 ± 3.8 (4/9)	44.7 ± 10.1 (6/9)	43.8, 46.2 (2/9)	>60.0 (0/9)	10^−8^
		0.003%	29.9 ± 12.0 ** (8/9)	21.8 ± 7.6 (9/9)	37.5 ± 13.8 (9/9)	30.8 ± 8.9 (6/9)	33.4 ± 0.4 (3/9)	32.7, 57.7 (2/9)	<10^−9^
		0.0003%	10.0 ± 1.6 (9/9)	23.6 ± 9.0 (9/9)	29.4 ± 6.7 (8/9)	37.1 ± 6.2 (9/9)	46.1 ± 9.1 (8/9)	56.0 ± 4.7 (4/9)	<10^−9^
	PS	0.007%	>60.0 (0/9)	38.3 ± 9.8 ** (9/9)	49.1 ± 8.3 ** (8/9)	52.6 ± 3.5 ** (6/9)	46.1 ± 11.7 (3/9)	54.2 (1/9)	<10^−9^
		0.0003%	11.6 ± 4.2 (9/9)	23.0 ± 11.1 (8/9)	31.9 ± 12.6 (9/9)	41.6 ± 12.3 (6/9)	48.0 ± 10.8 (4/9)	>60.0 (0/9)	10^−8^
	PC + PE + PS		>60.0 (0/9)	>60.0 (0/9)	>60.0 (0/9)	>60.0 (0/9)	>60.0 (0/9)	>60.0 (0/9)	>10^−4^
	SM	0.007%	>60.0 (0/9)	>60.0 (0/9)	>60.0 (0/9)	>60.0 (0/9)	>60.0 (0/9)	>60.0 (0/9)	>10^−4^
		0.0013%	>60.0 (0/9)	>60.0 (0/9)	>60.0 (0/9)	>60.0 (0/9)	>60.0 (0/9)	>60.0 (0/9)	>10^−4^
		0.0003%	25.3 ± 12.2 * (9/9)	36.9 ± 8.5 ** (7/9)	>60.0 (0/9)	51.2 ± 3.3 ** (3/9)	37.2 (1/9)	>60.0	10^−5^

^1^ PC: phosphatidylcholine, PE: phosphatidylethanolamine, PS: phosphatidylserine, and SM: sphingomyelin. PC + PE + PS indicates mixture of physiological concentrations of PC, 1PE, and PS. ^2^ Final concentrations in reaction mixture. In addition to physiological lipid concentrations of each lipid in human brain [29], 0.002% for PC, 0.003% for PE, 0.0003% for PS, and 0.0013% for SM, concentrations of total phospholipids (0.007% (highest)) and PS (0.0003% (lowest)) were tested. Concentration of PE in parenthesis indicates incomplete solubilization of PE in PBS. ^3^ Mean ± SD were calculated using wells that were determined to be positive out of the total numbers of wells used in three independent experiments with triplicate wells. Asterisks indicate log phase(s) with significant prolongation compared to the corresponding dilution of PBS-diluted seed. Statistical analyses were performed by Mann-Whiteney’s *U*-test for non-normal distribution or by *F*-test to assess variance levels, followed by Welch’s *t*-test for normal distribution. *: *p* < 0.05, **: *p* < 0.01.

## Data Availability

The original contributions presented in this study are included in the article/Supplementary Materials. Further inquiries can be directed to the corresponding author.

## Supplementary Materials

The following supporting information can be downloaded at: https://www.mdpi.com/article/10.3390/pathogens15030333/s1, Supplementary Material and Methods; Figure S1: Schematic illustration of expression system for rShPrP and mutants of rCerPrP; Figure S2: Detection of proteinase K-resistant PrP in brain homogenates from prion-affected animals; Figure S3: Effects of lipid extraction with EtOH on RT-QuIC reactions; Figure S4: Comparison of total PrP and protein levels before and after single-step BuMe extraction; Table S1: Summary of sheep and cattle used in this study; Table S2: RT-QuIC reactions of mouse-adapted scrapie prions using rodent rPrPs. References [20,25,26,27,52,53,54] are cited in the Supplementary Text.

## Abbreviations

The following abbreviations are used in this manuscript:

CJD	Creutzfeldt–Jacob disease
CWD	Chronic Wasting Disease
BSE	Bovine Spongiform Encephalopathy
PrP^Sc^	Abnormal Isoform of Prion Protein
PrP^C^	Cellular Isoform of PrP
CNS	Central Nervous System
C-BSE	Classical BSE
L-BSE	L-type Atypical BSE
RT-QuIC	Real-Time Quaking-Induced Conversion
ThT	Thioflavin T
rCerPrP	Recombinant Cervid PrP
NBH	Normal Brain Homogenate
C-scrapie	Classical Scrapie
A-scrapie	Atypical Scrapie
BuOH	2-Butanol
EtOH	Ethanol
MeOH	Methanol
PC	Phosphatidylcholine
PE	Phosphatidylethanolamine
PS	Phosphatidylserine
SM	Sphingomyelin
PBS	Phosphate-Buffered Saline
rMoPrP	Recombinant Mouse PrP
rHaPrP	Recombinant Hamster PrP
rBvPrP	Recombinant Bank Vole PrP
aa	a mino acid
rShPrP	Recombinant Sheep PrP
GdnHCl	Guanidine Hydrochloride
CV	Column Volumes
BuMe (3:1)	3:1 Mixture of BuOH and MeOH
SD	Standard Deviation
PMCA	Protein Misfolding Cyclic Amplification
Bo	Bovine
Sh	Sheep
Hu	Human
fg	Femtogram
