# Supplementary material for: Practical Real-Time Quaking-Induced Conversion for Detecting Classical Bovine Spongiform Encephalopathy and Classical and Atypical Scrapie Prions
Source: Pathogens. 2026 Mar 20;15(3):333. doi: 10.3390/pathogens15030333 (PMC13028819; doi:10.3390/pathogens15030333)
Supplement: Supplementary file 1 [file pathogens-15-00333-s001.zip › pathogens-4172168-supplementary.pdf]

Documents for Supporting Information

Supplementary Material and Methods

Reagents and chemicals

Anti-PrP mAb T2 (epitope: discontinuous region comprising MoPrP aa 132–217) conjugated with horseradish peroxidase (HRP) [1] was used for direct detection of PrP, whereas anti-PrP monoclonal antibody P4 (epitope: ShPrP aa 89–104) [2] was used for indirect detection of A-scrapie prions. Anti-Mouse IgG (Fab specific)-Peroxidase antibody produced in goat (Sigma-Aldrich) was used as a secondary antibody. The 2D-Silver Stain II (Cosmo Bio, Japan) was used for silver staining of SDS-PAGE gels.

Brain materials

Ten percent brain homogenates of C-BSE-affected cattle [3], C-scrapie-affected sheep [4], and A-scrapie-affected sheep were used for immunoblot analysis and as seeds for RT-QuIC assays.

Lipid extraction using EtOH

Endogenous lipids were extracted from 10% NBH using EtOH [5]. Briefly, 100 μl of 10% Mo NBH was mixed with 400 μl of EtOH and incubated for 5 min at room temperature. Insoluble components, including proteins and nucleotides, were precipitated by centrifugation at 15,000 × g for 5 min at room temperature. The supernatant, containing lipid components, was transferred to a new tube. The pellet was resuspended in 50 μl of PBS and mixed with 450 μl of EtOH for up to six sequential extractions. Supernatants from each extraction cycle were pooled. Pellets obtained after one, three, and six extraction cycles were resuspended in 100 μl of PBS (equivalent to 10% NBH). EtOH in pooled supernatants was evaporated at 37˚C, and the remaining lipids were resuspended into 100 μl of EtOH (equivalent to 10% NBH). Reconstituted pellet and supernatant fractions were stored at −30˚C and diluted with PBS to prepare is 2% equivalent samples, which were used as diluent for RT-QuIC seeds.

Immunoblotting

Immunoblot analysis was performed as described previously [6,7].

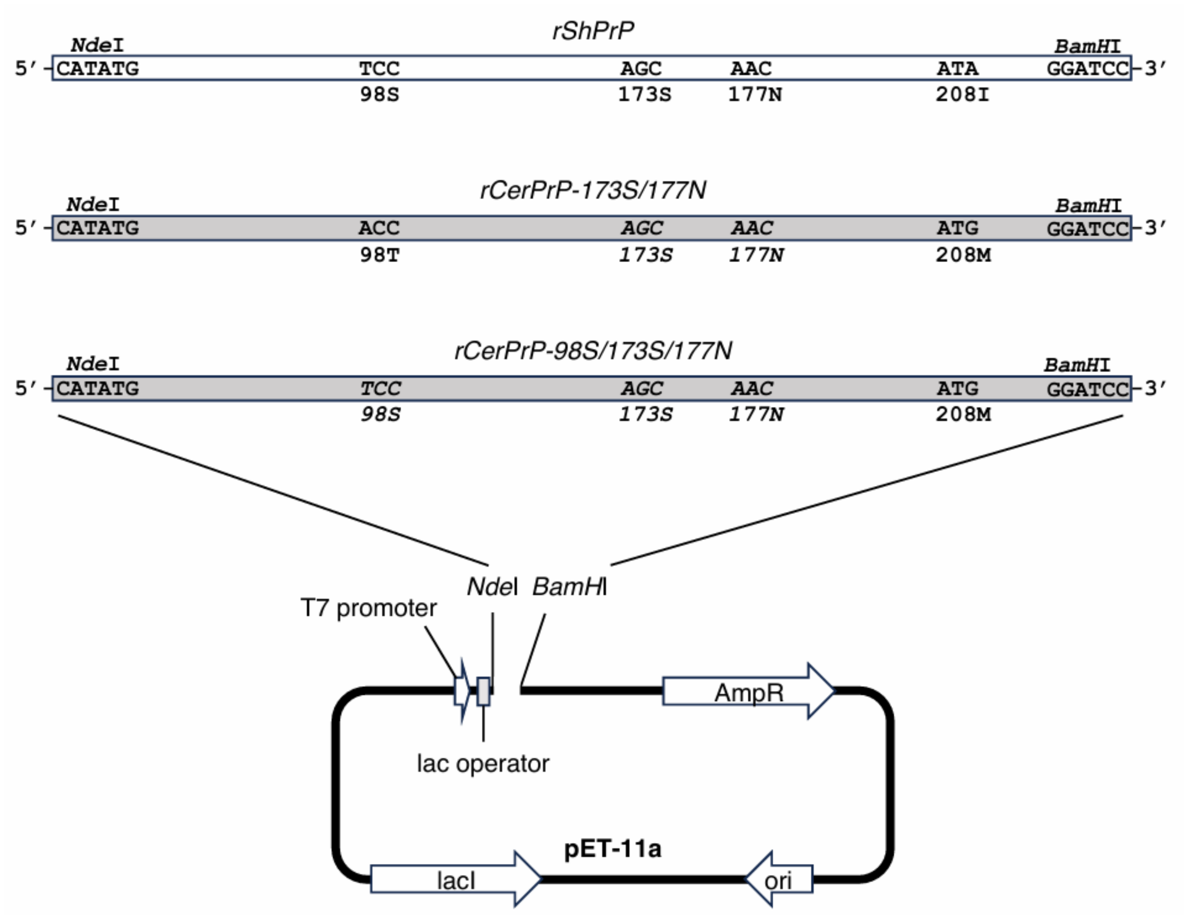

Figure S1. Schematic illustration of expression system for rShPrP and mutants of rCerPrP. Genes coding full-length rShPrP (genotype; $A_{136}R_{154}Q_{171}$) and rCerPrP (genotype; $G_{96}M_{132}S_{225}Q_{226}$) were amplified from the corresponding genomic DNA as described previously [6]. Genes coding rCerPrP-173S/177N were generated using assembly PCR as described previously [6]. The gene coding rCerPrP-98S/173S/177N was generated by replacing the T98 codon as shown in Material Methods. Each gene fragment was inserted into *NdeI* and *BamHI* sites of pET-11a (Novagen, USA). Gene backbones coding rShPrP and rCerPrP were represented by white and gray bars, respectively. Codons coding authentic amino acids of each species are indicated in Roman letters, whereas the codons replaced to ShPrP-specific codons by gene engineering are indicated in italic letters. Nucleotide sequences shown at 5′- and 3′-terminals indicated restriction enzyme recognition sequences for *NdeI* and *BamHI*, respectively.

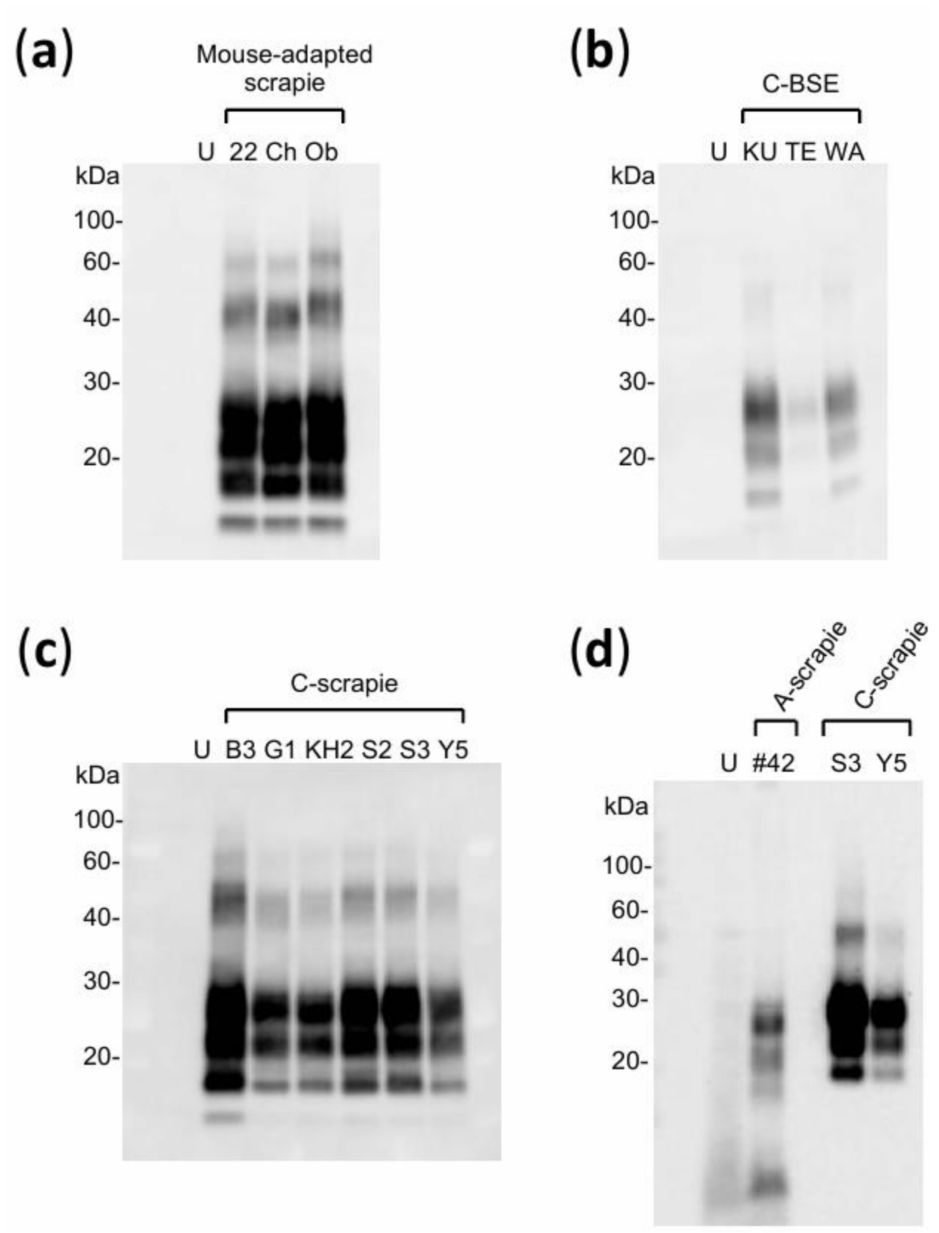

Figure S2. Detection of proteinase K-resistant PrP in brain homogenates from prion affected animals. (a) Prion-infected mice (22, Ch, and Ob: 22L, Chandler, and Obihiro strains, respectively), (b) naturally occurring C-BSE affected cattle in Japan (KU, TE, and WA), (c) C scrapie affected sheep (B3 and G1, experimentally infected; KH2, S2, S3, and Y5, naturally occurring cases in Japan), and (d) A-scrapie-affected sheep (#42). U: uninfected brain tissue from each species. Proteinase-K resistant PrP (PrP-res) from 2 mg (22L, Chandler, Obihiro, C BSE, and C-scrapie) and 10 mg (A-scrapie) of brain tissue equivalents was detected directly using T2-HRP [1] or indirectly using P4 [2]. Molecular weight markers are shown on the left in kDa.

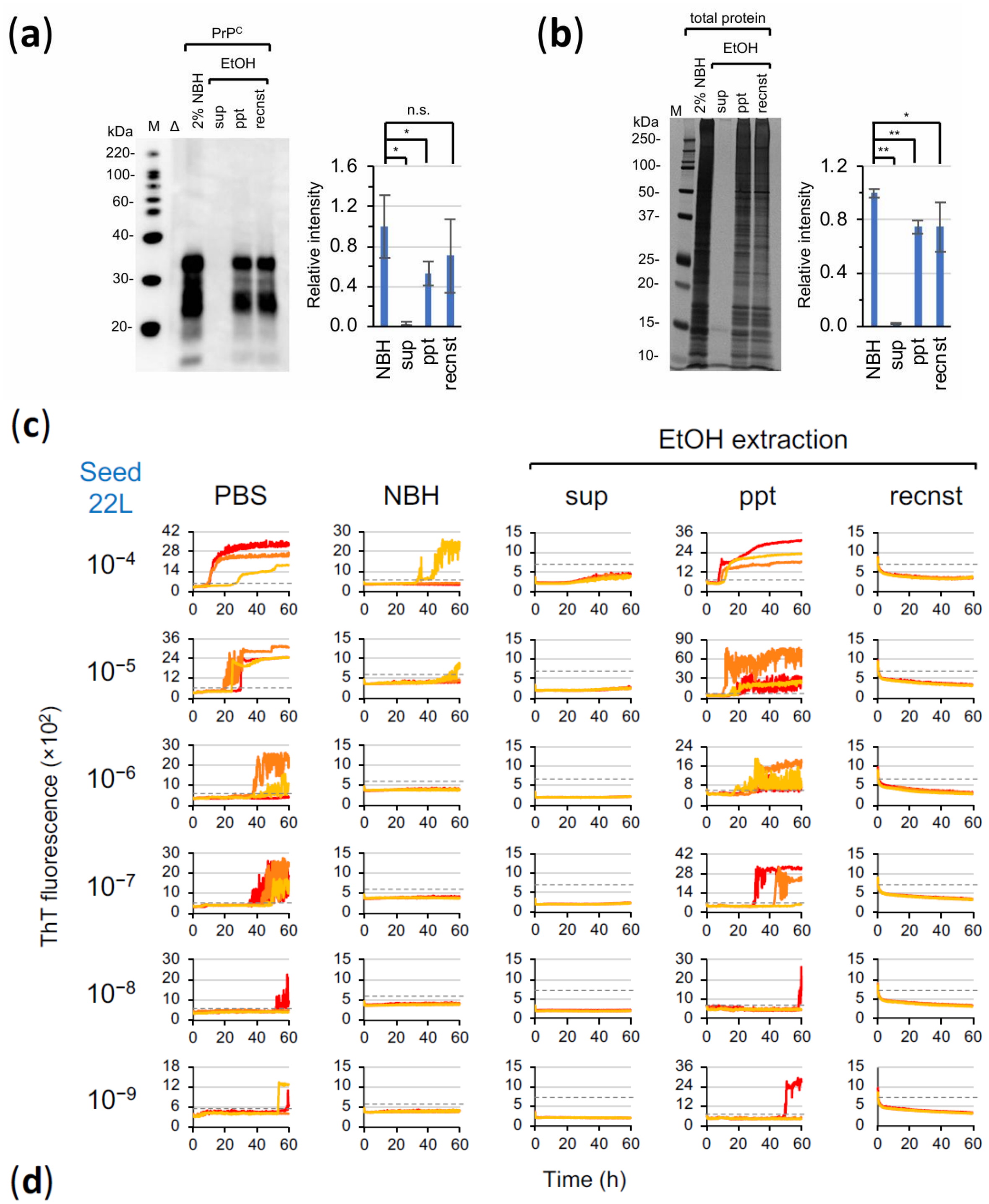

(d)

Seed	Diluent			Lag phase (h) 10^{-4}	10^{-5}	10^{-6}	10^{-7}	10^{-8}	10^{-9}	End-point
22L	PBS			13.5 ± 5.4	23.8 ± 6.6	30.8 ± 12.2	37.7 ± 14.1	51.8 ± 9.4	52.2 ± 10.5	<10^{-9}
	Mo NBH			34.0	52.8	>60.0	>60.0	>60.0	>60.0	10^{-5}
	EtOH	×1	sup	54.5 ± 3.2**	>60.0	54.4 ± 3.7**	>60.0	>60.0	>60.0	10^{-4}
			ppt	9.6 ± 1.1	15.5 ± 7.4	17.6 ± 5.1	40.1 ± 7.0	>60.0	>60.0	10^{-7}
		×3	sup	>60.0	>60.0	>60.0	>60.0	>60.0	>60.0	>10^{-4}
			ppt	9.6 ± 1.5	17.0 ± 6.6	25.2 ± 15.4	43.8 ± 8.4	47.6 ± 3.2	>60.0	10^{-8}
		×6	sup	>60.0	>60.0	>60.0	>60.0	>60.0	>60.0	>10^{-4}
			ppt	8.4 ± 1.2	18.9 ± 12.7	28.7 ± 5.1	41.1 ± 5.5	58.1 ± 0.4	49.5	<10^{-9}
			recnst	>60.0	>60.0	>60.0	>60.0	>60.0	>60.0	>10^{-4}

Figure S3. Effects of lipid extraction with EtOH on RT-108 QuIC reactions. Immunoblot detection of PrP^C (a) and sliver staining for total protein (b) in EtOH-extracted supernatant (sup), precipitated (ppt), and reconstituted fraction (recnst) after six sequential EtOH extractions. Bar graphs on the right indicate relative PrP^C (a) and protein levels (b) normalized to 2% mouse NBH (mean ± SD, n = 3). Delta (Δ) indicates blank lane. *$p < 0.05$ and **$p < 0.01$ by Student's *t*-test. (c) Representative RT-QuIC amplification curves for detection of 22L prions using rMoPrP after six sequential lipid extraction with EtOH. Seeds were serially diluted with PBS, 2% mouse NBH, extracted supernatant (sup), and precipitate (ppt), and reconstituted fraction (recnst). Dotted lines indicate reaction thresholds. (d) Lag phases (mean ± SD) and detection endpoints after one, three, and six sequential EtOH lipid extraction cycles are summarized. Asterisks indicate that lag phase(s) was significantly prolonged compared to the corresponding dilution of PRB-diluted seed. The statistical analyses were performed by a Mann-Whiteney's *U*-test for non-normal data, or by an *F*-test to assess variance levels followed by a Welch's *t*121. test for normal data. *: $p < 0.05$, **: $p < 0.01$.

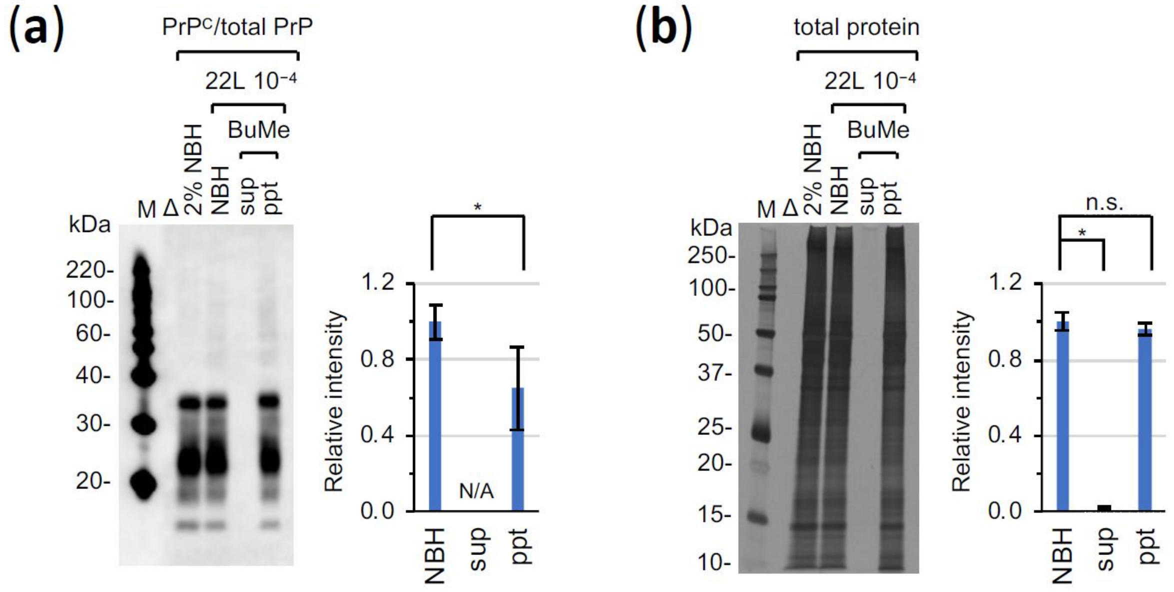

Figure S4. Comparison of total PrP and protein levels before and after single-step BuMe extraction. Immunoblot detection of total PrP (a) and sliver staining of total protein (b) in 2% mouse NBH (2% NBH), brain homogenates of 22L strain-infected mice diluted to 10^{-4} (22L 10^{-4}) with 2% mouse NBH (NBH), and corresponding reconstituted supernatant (sup) and precipitate (ppt) fractions following single-step lipid extraction with a 3:1 mixture of BuOH and MeOH (BuMe [3:1]). Ten micrograms of brain tissue equivalent were loaded per lane. Molecular weight markers on the left are in kDa. Bar graphs indicate relative intensities normalized to 22L strain-infected brain homogenates diluted to 10^{-4} with 2% mouse NBH (NBH). Δ: blank lane; N/A: background level signals that could not be quantified. *$p < 0.05$ and **$p < 0.01$ by Student's *t*-test.

Table S1. Summary of sheep a 143 nd cattle used in this study.

Disease	ID	Animal	Natural/ experimental	Age (month)	Symptom(s)	Confirmation [1)]	PrP-res amount [3)] (ng 0.01% BH/ml)
C-BSE	KUS	Cow	Natural	73	Left forelimb nerve paralysis, dyssyasia	WB, IHC	142.4 ng
	TE	Cow	Natural	67	NA [2)]	WB, IHC	35.6 ng
	WA	Cow	Natural	83	Dysstasia	WB, IHC	70.8 ng
Cscrapie	B3	Sheep	Experimental	21	Ataxia	WB	122.9 ng
	G1	Sheep	Experimental	26	Ataxia, debilitation	WB	70.1 ng
	KH2	Sheep	Natural	48	NA [2)]	WB	64.3 ng
	S2	Sheep	Natural	21	Ataxia, hypersensitivity	WB	93.8 ng
	S3	Sheep	Natural	16	Ataxia, hypersensitivity	WB	85.0 ng
	Y5	Sheep	Natural	41	Ataxia, debilitation	WB	57.4 ng
Ascrapie	#42	Sheep	Experimental	NA [2)]	NA [2)]	WB	1.8 ng

[1] WB, Western blotting; IHC, immunohistochemistry

[2] No information was available.

[3] Amount of PrP-res in 1 ml of 0.01% brain homogenates (BH).

Table S2. RT-QuIC reactions of mouse adapted s 150 crapie prions using rodent rPrPs.

Seed	rPrP	Diluent	Lag phase (h) [2]						Endpoint
			10^{-4}	10^{-5}	10^{-6}	10^{-7}	10^{-8}	10^{-9}	
22L	Mo	PBS	13.5 ± 5.4	23.8 ± 6.6	28.3 ± 5.6	34.9 ± 11.3	47.6 ± 9.0	46.0 ± 10.5	$<10^{-9}$
		NBH [1)]	34.0	52.8	>60.0	>60.0	>60.0	>60.0	10^{-5}
	Bv	PBS	9.3 ± 1.8	12.9 ± 1.9	19.3 ± 3.5	31.0 ± 9.4	45.6 ± 3.6	>60.0	10^{-8}
		NBH [1)]	>60.0	>60.0	>60.0	>60.0	>60.0	>60.0	$>10^{-4}$
	Ha	PBS	12.6 ± 3.1	24.2 ± 7.4	33.4 ± 11.5	39.7 ± 11.4	>60.0	>60.0	10^{-7}
		NBH [1)]	57.8	>60.0	>60.0	>60.0	>60.0	>60.0	10^{-4}
Chandler	Mo	PBS	14.3 ± 4.0	25.3 ± 11.4	38.1 ± 11.8	41.6 ± 10.5	53.2 ± 9.0	55.3 ± 8.4	$<10^{-9}$
		NBH [1)]	>60.0	>60.0	>60.0	>60.0	>60.0	>60.0	$>10^{-4}$
	Bv	PBS	19.2 ± 2.2	26.6 ± 9.4	24.9 ± 2.5	31.7 ± 3.7	38.2 ± 10.0	53.8 ± 7.1	$<10^{-9}$
		NBH [1)]	>60.0	>60.0	>60.0	>60.0	>60.0	>60.0	$>10^{-4}$
	Ha	PBS	30.1 ± 13.0	35.5 ± 9.4	34.2 ± 5.2	50.7 ± 7.1	53.5	>60.0	10^{-8}
		NBH [1)]	>60.0	>60.0	>60.0	>60.0	>60.0	>60.0	$>10^{-4}$
Obihiro	Mo	PBS	19.0 ± 5.5	27.8 ± 2.2	34.9 ± 4.9	49.3 ± 9.1	57.1 ± 4.9	>60.0	10^{-8}
		NBH [1)]	>60.0	>60.0	>60.0	>60.0	>60.0	>60.0	$>10^{-4}$
	Bv	PBS	20.5 ± 5.9	26.6 ± 11.0	35.8 ± 9.9	43.4 ± 8.5	57.7	41.5	$<10^{-9}$
		NBH [1)]	>60.0	>60.0	>60.0	>60.0	>60.0	>60.0	$>10^{-4}$
	Ha	PBS	26.2 ± 6.4	44.5 ± 9.9	50.4 ± 8.9	50.2	>60.0	>60.0	10^{-7}
		NBH [1)]	>60.0	>60.0	>60.0	>60.0	>60.0	>60.0	$>10^{-4}$

[1] Final concentrations of NBH (mouse NBH) in the 100 μl reaction mix was 0.1% (corresponding to the dilution at 10^{-3}).

[2] Mean ± SD from three independent experiments with three replicates were shown. Asterisks indicate that lag phase(s) after treatment was significantly prolonged compared to the corresponding dilution of PBS-diluted seed. The statistical analyses were performed by a Mann-Whiteney's *U*-test for non-normal distribution, or by an *F*-test to assess variance levels followed by a Welch's *t*-test for normal distribution. $*p < 0.05$, $**p < 0.01$

References

1. Shimizu, Y.; Kaku-Ushiki, Y.; Iwamaru, Y.; Muramoto, T.; Kitamoto, T.; Yokoyama, T.; Mohri, S.; Tagawa, Y. A novel anti-prion protein monoclonal antibody and its single chain fragment variable derivative with ability to inhibit abnormal prion protein accumulation in cultured cells. *Microbiol. Immunol.* **2010**, *54*, 112-121, doi:10.1111/j.1348-0421.2009.00190.x.
2. Harmeyer, S.P., E.; Groschup, M. H. Synthetic peptide vaccines yield monoclonal antibodies to cellular and pathological prion proteins of ruminants. *J. Gen. Virol.* **1998**, *79*, 937-945.
3. Shindoh, R.; Kim, C.L.; Song, C.H.; Hasebe, R.; Horiuchi, M. The region approximately between amino acids 81 and 137 of proteinase K-resistant PrPSc is critical for the infectivity of the Chandler prion strain. *J. Virol.* **2009**, *83*, 3852-3860, doi:10.1128/JVI.01740-08.
4. Horiuchi, M.; Nemoto, T.; Ishiguro, N.; Furuoka, H.; Mohri, S.; Shinagawa, M. Biological and biochemical characterization of sheep scrapie in Japan. *J. Clin. Microbiol.* **2002**, *40*, 3421-3426, doi:10.1128/JCM.40.9.3421-3426.2002.
5. Hoover, C.E.; Davenport, K.A.; Henderson, D.M.; Zabel, M.D.; Hoover, E.A. Endogenous Brain Lipids Inhibit Prion Amyloid Formation In Vitro. *J. Virol.* **2017**, *91*, doi:10.1128/JVI.02162-16.
6. Suzuki, A.; Sawada, K.; Yamasaki, T.; Denkers, N.D.; Mathiason, C.K.; Hoover, E.A.; Horiuchi, M. Involvement of N- and C-terminal region of recombinant cervid prion protein in its reactivity to CWD and atypical BSE prions in real-time quaking-induced conversion reaction in the presence of high concentrations of tissue homogenates. *Prion* **2020**, *14*, 283-295, doi:10.1080/19336896.2020.1858694.
7. Uryu, M.; Karino, A.; Kamihara, Y.; Horiuchi, M. Characterization of prion susceptibility in Neuro2a mouse neuroblastoma cell subclones. *Microbiol. Immunol.* **2007**, *51*, 661-669, doi:10.1111/j.1348-0421.2007.tb03954.x.
